# Structure of the vasopressin hormone–V2 receptor–β-arrestin1 ternary complex

**DOI:** 10.1126/sciadv.abo7761

**Published:** 2022-09-02

**Authors:** Julien Bous, Aurélien Fouillen, Hélène Orcel, Stefano Trapani, Xiaojing Cong, Simon Fontanel, Julie Saint-Paul, Joséphine Lai-Kee-Him, Serge Urbach, Nathalie Sibille, Rémy Sounier, Sébastien Granier, Bernard Mouillac, Patrick Bron

**Affiliations:** ^1^ CBS (Centre de Biologie Structurale), Université de Montpellier, CNRS, INSERM, Montpellier, France.; ^2^ Institut de Génomique Fonctionnelle, Université de Montpellier, CNRS, INSERM, 34094 Montpellier Cedex 5, France.

## Abstract

Arrestins interact with G protein–coupled receptors (GPCRs) to stop G protein activation and to initiate key signaling pathways. Recent structural studies shed light on the molecular mechanisms involved in GPCR-arrestin coupling, but whether this process is conserved among GPCRs is poorly understood. Here, we report the cryo–electron microscopy active structure of the wild-type arginine-vasopressin V2 receptor (V2R) in complex with β-arrestin1. It reveals an atypical position of β-arrestin1 compared to previously described GPCR-arrestin assemblies, associated with an original V2R/β-arrestin1 interface involving all receptor intracellular loops. Phosphorylated sites of the V2R carboxyl terminus are clearly identified and interact extensively with the β-arrestin1 N-lobe, in agreement with structural data obtained with chimeric or synthetic systems. Overall, these findings highlight a notable structural variability among GPCR-arrestin signaling complexes.

## Teaser

The structure of the vasopressin–V2 receptor–β-arrestin1 complex provides original insights into GPCR-arrestin architecture.

## INTRODUCTION

The biological role of arrestins in G protein–coupled receptor (GPCR) regulation was first found in the visual system more than 40 years ago, when arrestin1 was shown to bind to the light-activated rhodopsin, resulting in the inhibition of receptor signaling (*1*, *2*). The first nonvisual arrestin, found and characterized as a regulator of the β_2_-adrenergic receptor (β_2_AR) function (*3*), was named β-arrestin and then β-arrestin1 (βarr1, or arrestin2). Since then, a wealth of studies has defined the functions of arrestins in regulating GPCR desensitization, endocytosis, and intracellular trafficking (*4*, *5*). Beyond these roles, arrestins have been involved in the control of multiple cellular signaling pathways as scaffolding proteins (*6*). One of their better-understood functions is to activate mitogen-activated protein kinases (MAPKs), associated with cell cycle regulation, cell growth, and differentiation (*7*). Although it is currently accepted that MAPK activation requires endocytosis of stable GPCR-βarr complexes, it was recently shown that βarrs can drive MAPK signaling from clathrin-coated structures (CCSs) after GPCR dissociation (*8*). This “at a distance” βarr activation, in which transient engagement of the GPCR acts catalytically, also requires a series of interactions with membrane phosphoinositides and CCS-lattice proteins (*9*).

The molecular mechanisms and the cellular function of GPCR-βarr interactions were particularly well studied using the arginine-vasopressin (AVP) V2 receptor (V2R), which is involved in the control of water reabsorption and urine concentration in the kidney (*10*, *11*). This archetypal model system is well suited to analyze GPCR-βarr assembly because of a long-lasting and stable interaction. The V2R binds both βarr1 and βarr2 (or arrestin 3) with similar high affinity (*12*). Moreover, βarrs remain associated with desensitized V2R during clathrin-mediated endocytosis, a phenomenon directly linked to specific clusters of phosphorylated residues in the receptor C-terminal tail (V2RCter). This sustained interaction was first shown to dictate the slow trafficking of the V2R, particularly its rate of dephosphorylation, recycling, resensitization, and/or degradation (*13*, *14*). More recently, this sustained interaction was proposed to enhance AVP-induced cyclic adenosine monophosphate (cAMP) signaling from internalized V2R within endosomes (*15*). The V2R system was also used to investigate how GPCR phosphorylation patterns can orchestrate arrestin conformations and arrestin-dependent distinct signaling pathways (*16*).

A synthetic fully phosphorylated Cter peptide of the V2R (V2Rpp) was shown to functionally and conformationally activate βarr1 (*17*), and its strong affinity was used to investigate the active-state structure of βarr1 using x-ray crystallography (*18*). The complex was captured in the presence of a synthetic antibody fragment, Fab30, and revealed at high resolution how the N-lobe of βarr1 accommodates the V2R peptide. βarr1 and V2Rpp make extensive contacts, primarily through charge complementarity interactions between phosphate moieties of V2Rpp and arginine/lysine residues of βarr1 (*19*). The high affinity of V2Rpp for βarr1 was also instrumental in determining the three-dimensional (3D) structures of both muscarinic M2 receptor (M2R)–βarr1 and β_1_AR-βarr1 complexes, as in both cases, the natural C termini of receptors were replaced with that of the V2R (*20*, *21*). Both complexes show a V2RCter location similar to that determined in the V2Rpp-βarr1-Fab30 complex.

Although the presence of a phosphorylated V2RCter was necessary for the structure determination of active βarr1 and several GPCR-βarr1 complexes, the structure of the native full-length V2R in complex with βarrs has not been reported yet. Here, we describe the cryo–electron microscopy (cryo-EM) structure of the AVP-bound wild-type human V2R in complex with a truncated form of human βarr1 stabilized by the single-chain variable fragment of Fab30 (ScFv30). Together with the recent structures of the active conformation of the AVP-bound V2R in complex with the G_s_ protein (*22*–*24*), these findings provide major molecular and structural information to better understand arrestin-GPCR interactions and V2R-associated signaling pathways.

## RESULTS AND DISCUSSION

### Cryo-EM structure determination of the complex

The AVP-V2R-βarr1ΔCT-ScFv30 complex and the cryo-EM grids were prepared as described in Materials and Methods (figs. S1 to S3). A dataset of 14,080 movies was recorded on a Titan Krios microscope for single-particle analysis. To avoid missing any particle, a large number of objects were picked up using two algorithms that take advantage of neural networks and training strategies and processed with Relion (see Materials and Methods and figs. S4A and S5). Iterative rounds of 2D classification revealed particle classes with clear secondary structural details like V2R transmembrane (TM) domains (fig. S5). A conventional analysis in Relion and cryoSPARC (fig. S4B) provided a cryo-EM map with a limited 6.28-Å resolution, likely because of the strong dynamics of the system that classical 3D variance analyses could not manage efficiently (movie S1). Thus, to tackle the heterogeneity of the protein complex, a subset of particles sorted from the best 2D class averages was imported into cryoSPARC (*25*, *26*) and subjected to an optimized workflow based on iterative cycles of two-model ab initio refinement (fig. S4C). The stack of particles selected at each round corresponds to the best-resolved model. The successive models presented the same overall structural organization of the AVP-V2R-βarr1ΔCT-ScFv30 complex (fig. S6). In the end, this process resulted in a subset of 27,637 particles that, once subjected to a nonuniform (NU) 3D refinement, allowed us to compute a density map with a global resolution [Fourier shell correlation (FSC) = 0.143] of 4.75 Å for the AVP-V2R-βarr1ΔCT-ScFv30 complex and a 4.2-Å global resolution density map (EMD-14223) for the V2RCter-βarr1ΔCT-ScFv30 after V2R and detergent micelle signal subtraction (fig. S7, A and C). The particles were then further curated, resulting in a subset of 8296 particles, which was subjected to a NU 3D refinement, yielding a cryo-EM map with a global resolution of 4.7 Å (EMD-14221) for the AVP-V2R-βarr1ΔCT-ScFv30 complex (fig. S7, A and C). Data collection and processing are summarized in table S1.

**Fig. 1.**
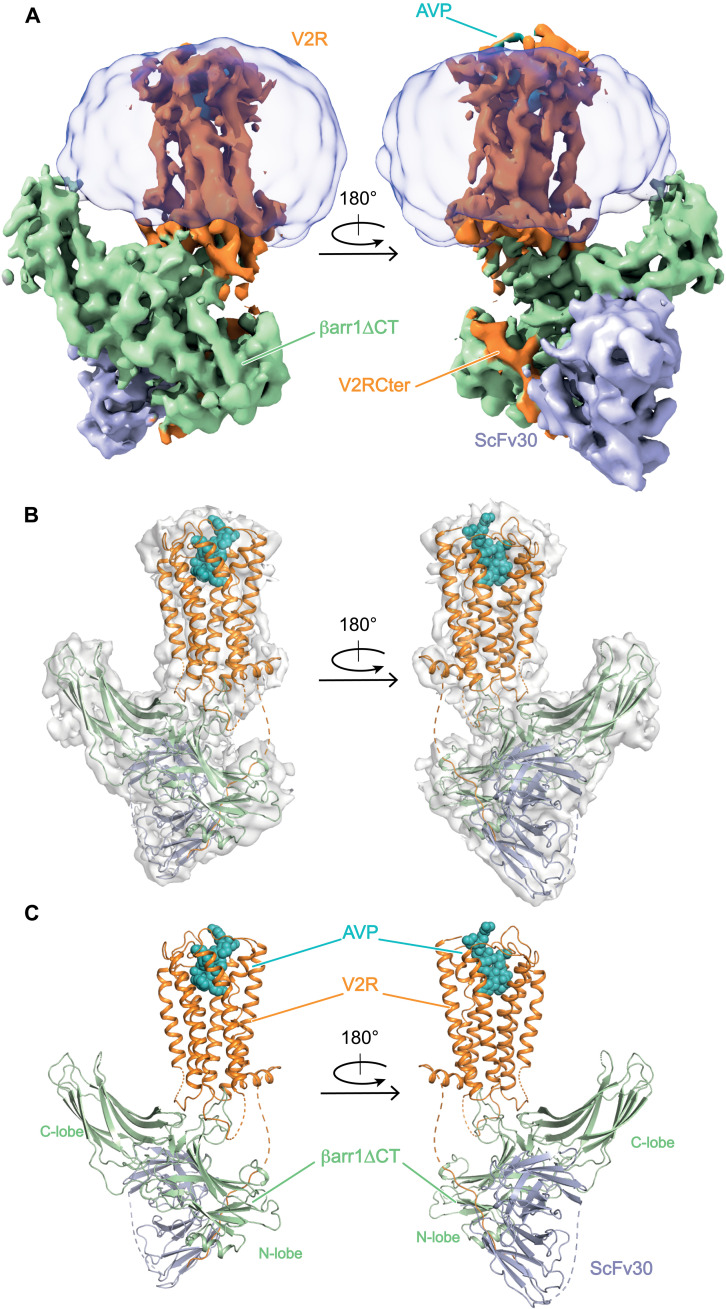
Overall architecture of the AVP-V2R-βarr1ΔCT-ScFv30 complex. (**A**) Orthogonal views of the cryo-EM density map of the complex. V2R and V2RCter are in orange, AVP is in cyan, βarr1ΔCT is in light green, ScFv30 is in gray-blue, and LMNG detergent micelle is in transparent gray. (**B**) Superimposition of the density map and the corresponding model of the complex. Missing parts in the protein chains are shown as dashed lines. The color scheme is as in (A). (**C**) Final 3D model of the complex.

The moderate resolution of reconstructions, therefore, indicates a high dynamical behavior of the AVP-V2R-βarr1ΔCT-ScFv30 complex around a preferential structural organization. However, the final EM maps that display local resolution ranging from 3.5 to 5.5 Å for the AVP-V2R-βarr1ΔCT-ScFv30 (fig. S7B) and the V2RCter-βarr1ΔCT-ScFv30 complexes (fig. S7D) allowed us to clearly determine the position and orientation of V2R, βarr1ΔCT, and ScFv30 and to model their backbones (Fig. 1). The N terminus (residues 1 to 31), parts of the intracellular loops (ICLs) (residues 148 to 156 from ICL1, residues 183 to 188 in ICL2, and residues 239 to 263 in ICL3), and the Cter of V2R (residues 343 to 355 and 369 to 371) are not included in the final model. The model of βarr1ΔCT includes residues 6 to 365 (except for residues 332 to 339), whereas ScFv30 is nearly complete (residues 110 to 128 are missing). AVP is also constructed in the refined model (Fig. 1). Refinement and validation statistics of the AVP-V2R-βarr1ΔCT-ScFv30 and V2RCter-βarr1ΔCT-ScFv30 structural models [Protein Data Bank (PDB) 7r0c and 7r0j, respectively] are summarized in table S1. The overall architecture of the AVP-V2R-βarr1ΔCT-ScFv30 complex (Fig. 1) is similar to the one previously described for muscarinic M2R–βarr1, adrenoceptor β_1_AR–βarr1, and neurotensin receptor 1 (NTSR1)–βarr1 complexes (*20*, *21*, *27*, *28*). The V2R seven-TM helical bundle engages the βarr1ΔCT in a core conformation [as defined in (*29*)], with its Cter contacting the βarr1ΔCT N domain and the ScFv30 (Fig. 1). The βarr1ΔCT interacts with V2R through its central crest region, whereas its hydrophobic C-edge inserts into the detergent micelle. The ScFv30 interacts with both βarr1ΔCT N- and C-lobes. The model revealed that both V2R and βarr1ΔCT present characteristics of active-state structures with specific features as discussed below.

### The βarr1 engages the V2R with an atypical orientation and a tilted conformation

**Fig. 2.**
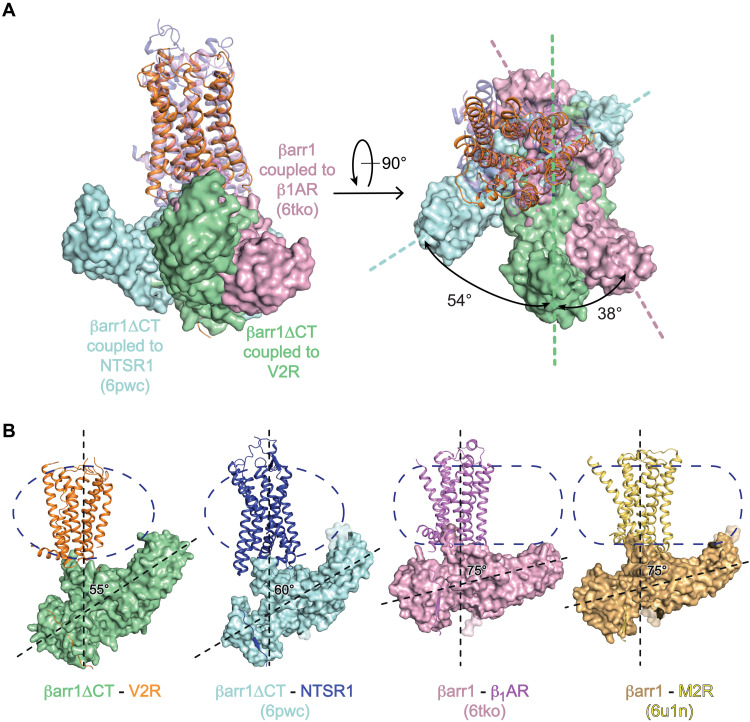
Atypical orientation of βarr1ΔCT and tilted conformation involving the C-edge domain. (**A**) Overlay of the V2R-βarr1ΔCT structure with NTSR1-βarr1ΔCT (6wpc) and β_1_AR-βarr1 (6tko) structures, on the basis of alignment of the receptor chains, viewed from the membrane (left) and from the extracellular space (right). The difference in the orientation of βarr1ΔCT in the complexes is given by the angle of rotation. V2R and βarr1ΔCT are colored as in Fig. 1, βarr1 and β_1_AR (6tko complex) are in pink, and βarr1ΔCT and NTSR1 (6pwc complex) are in blue and purple, respectively. (**B**) Comparison of the tilted conformations of βarr1 (or βarr1ΔCT) in the different GPCR complexes. The contacts between the βarr1 C-edge and the membrane-like environment are visible for each complex. The detergent micelles (V2R and NTSR1 complexes) or nanodiscs (β_1_AR and M2R complexes) are shown as dashed lines. The angle between the longitudinal axis of βarr1s and GPCRs is indicated for each complex. V2R and βarr1ΔCT are colored as in Fig. 1. NTSR1 (6pwc) is in dark purple, whereas βarr1ΔCT is in blue. βarr1 and β_1_AR (6tko complex) are in pink, and βarr1 and M2R (6u1n complex) are in gold and yellow, respectively.

From reported structures of GPCR-βarr1 complexes (*20*, *21*, *27*, *28*), βarr1 can harbor two main perpendicular orientations relative to the GPCR bundle axis, separated by a rotation of approximately 90° parallel to the membrane plane. One is observed for M2R and β_1_AR complexes and the other for NTSR1 complexes (Fig. 2A). Unexpectedly, the βarr1ΔCT presents an atypical intermediate position in the AVP-V2R-βarr1ΔCT-ScFv30 complex, being rotated by 54° or 38° when compared to the NTSR1-βarr1 or to the β_1_AR-βarr1 complex structures, respectively (Fig. 2A). A comparison with the other reported GPCR-βarr1 complexes further highlights the unique organization of the AVP-V2R-βarr1ΔCT-ScFv30 complex (fig. S8).

Besides the atypical orientation, the C-edge of the βarr1ΔCT C-lobe inserts into the detergent micelle (Fig. 1A), through a domain (the s18s19 loop, only partially seen in the density map of the complex) that has been shown to directly interact with clathrin (*30*). This interaction causes a strong tilt with a 55° angle between the longitudinal axis of βarr1ΔCT and V2R (Fig. 2B). βarr1 C-edge membrane anchoring has been observed in the structure of all known GPCR-βarr1 complexes (Fig. 2B), whatever the artificial membrane-like environment used for their purification and stabilization (detergent micelles of different chemical nature and nanodiscs). This phenomenon participates in the asymmetry of the different complexes and probably helps in stabilizing βarr1 interactions with GPCRs (*20*). A tilt comparable to the one determined in the AVP-V2R-βarr1ΔCT-ScFv30 structure has been observed in the cryo-EM structure of the NTS_8–13_-NTSR1-βarr1ΔCT complex (*27*), prepared with equivalent detergent micelles [a mix of lauryl maltose neopentyl glycol (LMNG), glyco-diosgenin (GDN), and cholesteryl hemisuccinate (CHS)], and in the presence of a phosphatidylinositol-4,5-bisphosphate [PtdIns(4,5)P_2_] analog, the dioctyl-PtdIns(4,5)P_2_ (diC8PIP2) (fig. S9A). In other complexes, in which diC8PIP2 is not present and where the lipid environment of the GPCRs is different (nanodiscs), the arrestin tilt is less pronounced, with an angle ranging from 60° (NTS_8–13_-BRIL-NTSR1-βarr1ΔCT complex in digitonin micelles) to 75° (M2R-βarr1 and β_1_AR-βarr1 complexes, both in nanodiscs) (Fig. 2B). The LMNG-GDN-CHS micelles are artificial membrane-like systems, and, although the βarr1ΔCT-V2R tilt may be amplified because of the small size of these micelles (average diameter is less than 10 nm) compared to a planar bilayer like the plasma membrane, this tilted orientation may reflect the importance of GPCR-arrestin interactions in the context of subcellular structures with a high degree of curvature like CCSs [around 100 nm; (*31*)] or endosomes (100 to 500 nm) from which GPCR-arrestin signaling has been demonstrated to occur (*8*, *9*). This hypothesis has been previously proposed for the NTSR1-βarr1ΔCT complex (*27*). We hypothesize that the more the membrane curvature is pronounced, the more the extent of βarr1 tilt would be increased.

It is worth noting that although the AVP-V2R-βarr1ΔCT-ScFv30 and the NTS_8–13_-NTSR1-βarr1ΔCT complexes were both prepared and purified in a Hepes-NaCl buffer with LMNG-GDN-CHS detergent micelles, the orientation of βarr1ΔCT relative to the GPCR bundle in these two assemblies is quite different (Fig. 2, A and B, and fig. S8). This implies that the βarr1ΔCT original orientation in the AVP-V2R-βarr1ΔCT-ScFv30 complex is mainly driven by a peculiar arrestin-GPCR interface, as discussed later in the manuscript.

A phosphoinositide/phosphoinositol binding site was identified in the C-lobe of βarr1/2 proteins (*32*) and was shown to play a key role in anchoring these GPCR signaling partners in the plasma membrane at CCSs. In βarr1/2, basic residues (K232, R236, and K250 versus K233, R237, and K251 in βarr1 and βarr2, respectively), likely to interact with negatively charged phosphates of phosphoinositides, participate in this binding site. Their mutation leads to βarr1/2 being unable to be recruited either to CCSs (*32*) or to the plasma membrane (*9*). A βarr1 variant containing these three mutations showed a 40% reduction in recruitment to NTSR1 when compared to the wild type (*27*). In the NTSR1-βarr1ΔCT complex, diC8PIP2 forms a bridge between the membrane side of NTSR1 TM1, TM2, and TM4 and the C-lobe of βarr1ΔCT (fig. S9).

While we added diC8PIP2 during the preparation of the cryo-EM samples, the density map resolution is not high enough to be sure of its correct positioning in the AVP-V2R-βarr1ΔCT-ScFv30 complex. We, however, modeled its putative localization based on several evidence (fig. S9, A and B). Our density map revealed an elongated density protruding from the detergent micelle in a position that would face the βarr1 phosphoinositide binding site. This is better viewed in the unsharpened map of the complex (fig. S9A). Moreover, when aligning the AVP-V2R-βarr1ΔCT-ScFv30 and NTS_8–13_-NTSR1-βarr1ΔCT (*27*) complexes onto the βarr1ΔCT protein, the diC8PIP2 moieties also superimpose (fig. S9B). Because of the atypical orientation of βarr1ΔCT relative to V2R, the phosphoinositide molecule, if positioned at this place, would bridge the C-lobe of βarr1ΔCT with the membrane side of V2R TM4 only (fig. S9B). We hypothesize that the presence of the phosphoinositide moiety may help the insertion of βarr1ΔCT in the detergent micelle and the stabilization of the V2R-βarr1ΔCT interactions, an effect that can be observed when comparing the structure of NTS_8–13_-NTSR1-βarr1ΔCT complex in the presence or absence of diC8PIP2 (two different types of detergent micelles, LMNG-GDN-CHS versus digitonin). The βarr1ΔCT tilt is more pronounced if the diC8PIP2 is incorporated in the complex (*27*, *28*). To analyze this potential stabilizing effect for the AVP-V2R-βarr1ΔCT complex, we performed molecular dynamics (MD) simulations. We found that in the presence of diC8PIP2, root mean square deviations and fluctuations of βarr1ΔCT Cα carbons were obviously reduced (fig. S10). Moreover, the diC8PIP2 constrained βarr1ΔCT C-lobe in a tilted conformation (fig. S11). In addition, βarr1ΔCT exhibited a mobility (rotation and tilting) that was less pronounced in the presence of diC8PIP2 (figs. S10 and S11). In conclusion, the presence of diC8PIP2 in the sample combined with the insertion of the βarr1ΔCT C-edge into the detergent micelle are probably key parameters to enhance the stability of the AVP-V2R-βarr1ΔCT-ScFv30 complex.

### V2R and βarr1ΔCT display active conformational states

**Fig. 3.**
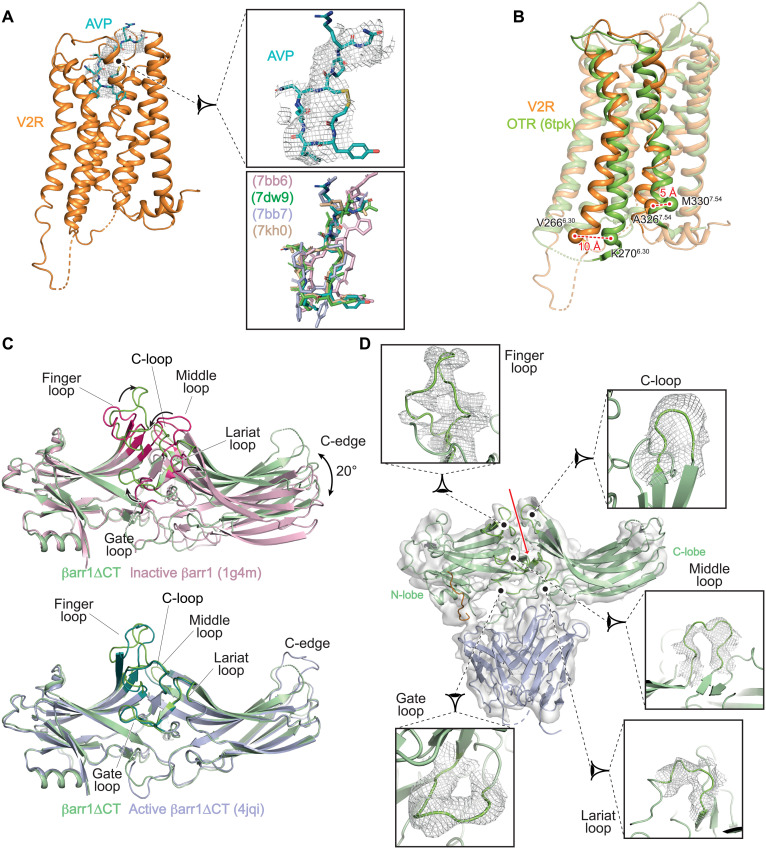
Active conformations of V2R and βarr1ΔCT. (**A**) AVP (blue in the model, density is shown as a mesh) binding to V2R (orange). A close-up view of the AVP binding pose is shown on the right (top). Overlapping of V2R-bound AVP in both G protein– and arrestin-associated complexes (bottom). (**B**) Comparison of the V2R structure with the inactive OTR (green) structure. Residues 6.30 and 7.54 (Ballesteros-Weinstein numbering) are chosen as references (V266 and A326 in V2R, K270, and M330 in OTR) for measuring the outward (10 Å) and inward (5 Å) movement of TM6 and TM7, respectively. (**C**) The βarr1ΔCT (pale green) in the V2R complex is superimposed onto the inactive (top) and active (bottom) states of the βarr1 (1g4m and 4jqi, respectively). Movements of the different loops (in raspberry in the inactive conformations) are indicated by arrows. The C-lobe is translated by 20° upon activation. Inactive βarr1 is illustrated in pink, and active βarr1ΔCT is in light blue. (**D**) Overlay of the density map and the corresponding model of the βarr1ΔCT with close-up views for the different active loops. The color scheme is identical to that of Figs. 1 and 2. Each panel displays the map density as a mesh and the 3D model as a ribbon. The red arrow indicates the central furrow between βarr1ΔCT N- and C-lobes.

Both the receptor and βarr1 displayed the main hallmarks of active conformations (Figs. 1 and 3). From the receptor point of view, a clear density is observed for the full agonist AVP at the top of the 7TM helix bundle adopting, at this level of resolution, a conformation close to those reported for the active AVP-V2R-G_s_ structures (Fig. 3A) (*22*–*24*). Both V2R TM6 and TM7 exhibited a displacement by 10 and 5 Å, respectively, when compared to their counterparts in the inactive antagonist-bound oxytocin receptor (OTR) structure (Fig. 3B) (*33*). These displacements are similar to those observed in the different structures of AVP-V2R-G_s_ complexes (*22*–*24*). From the βarr1 point of view, the superimposition of βarr1ΔCT with the inactive βarr1 (PDB 1g4m) clearly shows a rotation of the C-lobe of ~20° relative to the N-lobe (Fig. 3C) and movements of the finger loop (FL), gate loop (GL), middle loop (ML), lariat loop (LL), and C-loop (CL), as previously described (*34*). Moreover, the alignment of βarr1ΔCT to the active βarr1 (crystal structure of βarr1 in the presence of the V2Rpp phosphopeptide, PDB 4jqi) demonstrates that the FL, GL, ML, and LL also adopt an active state to shape a central crest necessary for GPCR coupling (Fig. 3C). Depending on the GPCR-βarr1 complex considered, different domains of βarr1 are affected. For instance, in the NTS_8–13_-NTSR1-βarr1ΔCT complex (*27*), the FL, ML, CL, and C-edge display conformations that are different from those in the βarr1 active structure. In the AVP-V2R-βarr1ΔCT, the receptor mainly affects the βarr1ΔCT FL, the CL, and the C-edge upon interaction, a similar situation found in the M2R-βarr1 complex (*20*). On the basis of the density map with a better global resolution of 4.2 Å, the different loops of the active form of βarr1ΔCT were confidently assigned and modeled (Fig. 3D). The conformation of FL, ML, and LL from N-lobe on one side and that of CL and β strand 16 from C-lobe on the other side circumscribe a specific furrow (Fig. 3D, red arrow), which takes a major part in the specific orientation between the receptor and the βarr1 (see below).

### The βarr1/V2R interaction surface defines a novel orientation for an arrestin-GPCR signaling complex

**Fig. 4.**
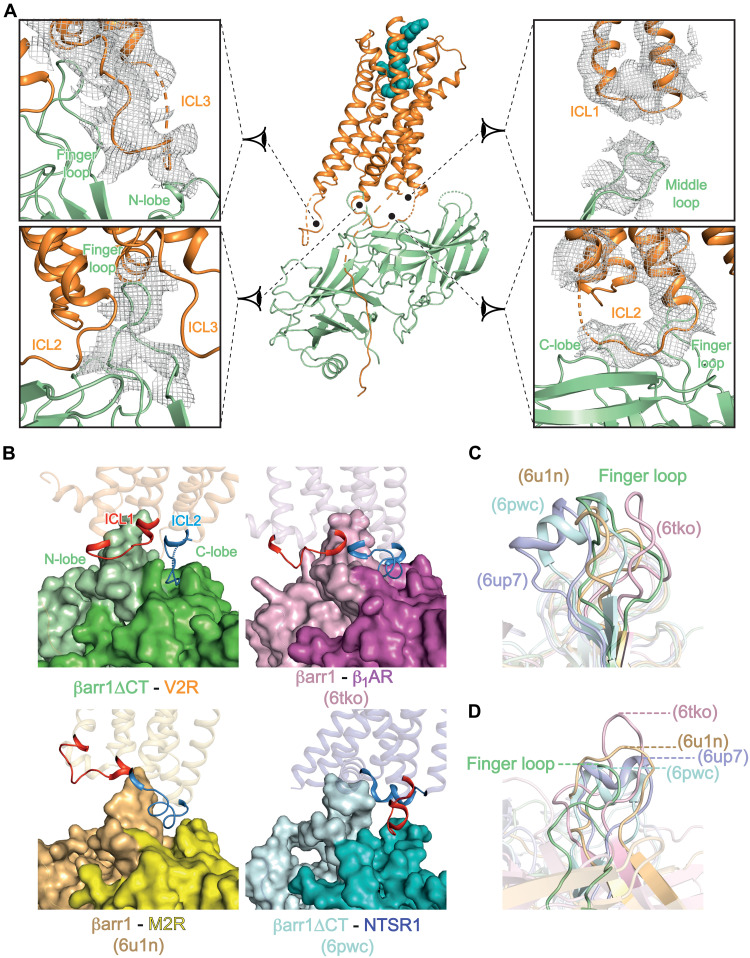
The V2R/βarr1ΔCT interface. (**A**) The model of the AVP-V2R-βarr1ΔCT complex is shown in the middle with close-up views for the different domains involved in the interaction surface. The color scheme is identical to that of Fig. 1. Each zoom panel displays the map density as a mesh and the corresponding model as a ribbon. (**B**) Position of ICL2 (or ICL1) in the central furrow. The V2R-, β_1_AR-, M2R-, and NTSR1 (PDB 6pwc)–associated complexes are compared. ICL1 is in red, and ICL2 is in blue. To have a better view of the central crevice, the N-lobe and the C-lobe of βarr1s are highlighted in light- and dark-related colors: green for the present V2R-βarr1ΔCT complex, pink for 6tko, yellow for 6un1, and blue for 6pwc. (**C** and **D**) Orthogonal views of the βarr1 FL in the different complexes. The color scheme is identical to that of Fig. 2. (C) Alignment made with βarr1s to illustrate the FL variable conformations in the different GPCR complexes. (D) Alignment made with GPCRs to highlight the FL insertion deepness.

The peculiar architecture of the AVP-V2R-βarr1ΔCT-ScFv30 complex results in an original V2R-βarr1 interface, as compared to those reported for other GPCR-βarr1 complexes. All ICLs and the 7TM cavity of the V2R are in contact with βarr1ΔCT (Fig. 4A and fig. S12). First, the density map reveals that ICL1 of V2R directly contacts the ML in the central crest of βarr1ΔCT (Fig. 4A). This V2R region has been previously shown to interact with the N-terminal helix of the G_s_ α subunit (*22*). Second, although ICL2 is not entirely seen in the density map, this V2R region (residues R139 to A147) notably binds in a particularly well-defined furrow between the N- and C-lobes of βarr1ΔCT, lying in a central position (Figs. 3D and 4, A and B, and fig. S12). Third, most of the V2R ICL3 is not resolved (residues 239 to 263 are missing); however, clear contacts are seen with the N-lobe of βarr1ΔCT, particularly with the N-terminal part of the FL (Fig. 4A). In addition, the ICL3 of V2R displays a cluster of arginine residues (RRRGRR at positions 247 to 252) that may also create ionic contacts with βarr1ΔCT negatively charged residues, for instance, E66, E67, D78, E134, D143, E145, E152, E155, and E156. To explore a potential role of this arginine cluster in βarr recruitment, we generated a V2R mutant that lacked this sequence [V2R-AAAGAA(247–252)] and found that it was as efficient as the wild-type V2R to recruit βarr2 (fig. S13) using an assay based on HTRF technology (see Materials and Methods). Using a chimeric mutagenesis approach, we and others previously demonstrated that the Cter of V2R is sufficient to transform the AVP V1B and V1A receptors from a transient to a long-lasting interaction in living cells (*14*, *35*), suggesting that this arginine motif in ICL3 by itself cannot play a role in a higher affinity of V2R for β-arrs. To explore further these potential interactions, we have also performed MD simulations of AVP-V2R-βarr1ΔCT including V2R ICL2 and ICL3. These two ICLs were highly mobile in the different simulations (fig. S11), consistent with the lack of visibility in the cryo-EM density maps. More pronounced potential contacts were only observed between the V2R arginine cluster and residues P131-G132 in the ML of βarr1 N-lobe in the presence of diC8PIP2, which reduced the mobility of the whole complex and of ICL3 (figs. S10 and S11 and table S2). We did not observe strong ionic interactions, confirming that the arginine cluster appears unlikely to be crucial for βarr binding.

Together, as compared to the structures of other GPCR-βarr1 complexes, the combination of interactions between ICLs of V2R and the βarr1ΔCT is quite singular (Fig. 4, Table 1, and fig. S14). As opposed to V2R, ICL1 from M2R does not interact at all with βarr1 (Fig. 4B and Table 1), while that of β_1_AR is positioned close to both the ML and part of FL but with a slightly different orientation (Fig. 4B, Table 1, and fig. S14). The ICL2s from β_1_AR or M2R also insert into the central furrow but with an orientation and deepness that are slightly different (Fig. 4B and Table 1). Unexpectedly for the NTSR1-βarr1 complexes, ICL1 binds to this furrow instead of ICL2 (Fig. 4B and Table 1), a specificity that may explain the particular rotation (~90°) of βarr1 in the NTSR1-βarr1 structure when compared to the β_1_AR and M2R complexes (Fig. 2A). The structures of β_1_AR-βarr1 and M2R-βarr1ΔCT complexes (*20*, *21*) show that residues in the β strand 16 (Y249), CL (I241), LL (R285), or FL (Y63 and R65) domains of βarr1 directly participate in the binding of ICL2 and consequently may affect the orientation between the receptor and βarr1 (Table 1). Notably, Y249 and R285 also interact with ICL1 in the NTSR1-βarr1 complexes (*27*, *28*). These results highlight the role of some key residues in βarr1, regardless of the region of GPCR ICLs they interact with. The global resolution is lower for the V2R-βarr1ΔCT complex; however, it is tempting to speculate that these residues are involved in the binding of V2R ICL2 (figs. S12 and S14). Together, these data suggest that, depending on the GPCR, the interactions between ICL1 and ICL2 with the particular central furrow of βarr1 are, at least in part, involved in determining the architecture of the signaling GPCR-βarr1 complexes.

**Table 1.** Receptor ICL-βarr1 interactions observed in different GPCR-βarr1 structures, in comparison with the rhodopsin-arrestin1 complex. Contact residues or domains from receptors and arrestins are indicated in bold and italics, respectively. For the V2R-βarr1ΔCT complex, only regions that are seen in the density map are indicated. For other GPCR-βarr1 complexes, residues involved in ICL-βarr1 contacts within a 4-Å distance are shown. In the 5w0p complex, mouse visual arrestin1 (*m-arr1*) is associated with human rhodopsin (*91*). For information, many residues of the βarr1 (for instance, Y63, Y249, and R285) that are always in contact with the different GPCRs (whatever the orientation) are conserved in the m-arr1 (with a different numbering; Y68, Y256, and R292).

**Receptor ICLs**	**V2R/β*arr1*Δ*CT* complex**	**β_1_AR/β*arr1* complex (6tko)**	**M2R/β*arr1* complex (6u1n)**	**NTSR1/β*arr1*Δ*CT* complex (6up7)**	**NTSR1/β*arr1*Δ*CT* complex (6pwc)**	**Rhodopsin/*m-arr1* complex (5w0p)**
**ICL1**	**R68-I74/** *ML, FL*	**T69-T74/** *E66-D67 (FL), T136 (ML)*	No contact	**S93-T100**/central furrow involving Y63 and R65 (FL), K284-G286 (LL), A247, Y249-V251* *(*b *strand 16)*	**S93-T100**/*central furrow involving R285*^†^ *(LL), Y249-C251*^†^ *(*b *strand 16)*	**H65-P71**/*I73 (FL)*
**ICL2**	**R139-A147**/*central furrow between N and C-lobes*	***S145-R155****/central furrow involving F61, Y63-R65 (FL), E134-T136 (ML), R285 (LL), I241, L243 (C-loop), Y249 (*β *strand 16)*	**K127-R135**/*central furrow involving Y63*^†^ *(FL), L129*^†^*, L140*^†^ *(ML), R285*^†^ *(LL), Y249 (*β *strand 16)*	**H172-R182/** *N245-T246 (C-loop)*	**H172-R182/***F244*^†^ *(C-loop)*	**C140-G149/***central furrow involving F66, Y68 (FL), L133-Q134, K142-C144 (ML), R292 (LL), V248, Y251 (C-loop) D254, Y256* *(*β *strand 16)*
**ICL3**	**Subdomain E231-S235**/*FL (residues at the junction with* β *strands 5 and 6)*	Not seen in the structure	Not seen in the structure	**Subdomain F286-P292/** *G72-K77 (C-ter of FL, at the junction with* β* strand 6)*	Not seen in the structure	**Q236-A241/** *R82-L84 (*β *strand 6), R319, M322 (between GL and* β *strand 18)*

*C251 was replaced with a valine in this cysteine-free variant of human βarr1 (*27*).

†Residues within a 5-Å distance.

Last, the V2R TM cavity formed by the outward motion of TM6 engages the FL of βarr1ΔCT (from Y63 to L73) (Fig. 4, C and D, and figs. S1B and S14). A comparison of the V2R-coupled βarr1ΔCT structure with those published for other complexes highlights a strong variability of FL, both in its conformation and its GPCR binding deepness (Fig. 4, C and D). In all GPCR-βarr1 complexes, the FL inserts the receptor core in a pocket delimited by TM2, TM3, TM6, and TM7 and establishes contacts with different residues from these TM helices. However, depending on the receptor, FL can adopt an α-helical domain (in the NTSR1-βarr1ΔCT complex) or can be deeply inserted in the GPCR core (in the β_1_AR-βarr1 complex), contacting conserved I^6.40^ or Y^7.53^ (Weinstein-Ballesteros nomenclature) (Fig. 4, C and D). At the V2R-βarr1ΔCT interface, the FL inserts V2R like in other GPCR-arrestin complexes but not as deep into the TM core as observed for βarr1 FL in β_1_AR (*21*). More particularly, it seems to contact residues from the cytoplasmic sides of TM2, TM3, and TM6. For comparison, an interaction between the tip of FL and R^3.50^ (Ballesteros-Weinstein nomenclature) in TM3 has been seen in the M2R-βarr1 (R121^3.50^ and βarr1 D69) and the β_1_AR-βarr1 (R139^3.50^ and βarr1 D69) complexes (*20*, *21*). Considering the V2R, the corresponding R137^3.50^ is part of the ionic lock motif and has been shown to directly interact with the free carboxylic acid function of the G_s_ protein α subunit Cter in the active structure of the AVP-V2R-G_s_-Nb35 complex (*22*). Therefore, in addition to the key ICL2-βarr1ΔCT interactions, additional structural parameters including ICL1 positioning, ICL3 dynamic interaction, and a particular FL conformation and binding deepness in the 7TM core are involved in defining the atypical orientation of βarr1ΔCT bound to the V2R.

### Constitutive phosphorylation of V2R and interaction with βarr1

The Cter of GPCRs plays a key role in many aspects of their regulation, through phosphorylation by various GPCR kinases (GRKs) and subsequent binding to arrestins. The number and arrangement of phosphates may vary substantially for a given receptor, and different phosphorylation patterns have been shown to trigger different arrestin-mediated effects (*36*). Using atomic-level simulations and site-directed spectroscopy in one study (*16*) or x-ray crystallography combined with Bioluminescence Resonance Energy Transfer (BRET) and ^1^H nuclear magnetic resonance in another (*37*), the structural basis regulating this phosphorylation barcode has been revealed recently, indicating how GPCR phosphorylation affects arrestin binding and conformation. Specific phosphobarcodes of V2R Cter phosphopeptides were correlated with conformational changes in βarr1 and selective downstream signaling responses.

**Fig. 5.**
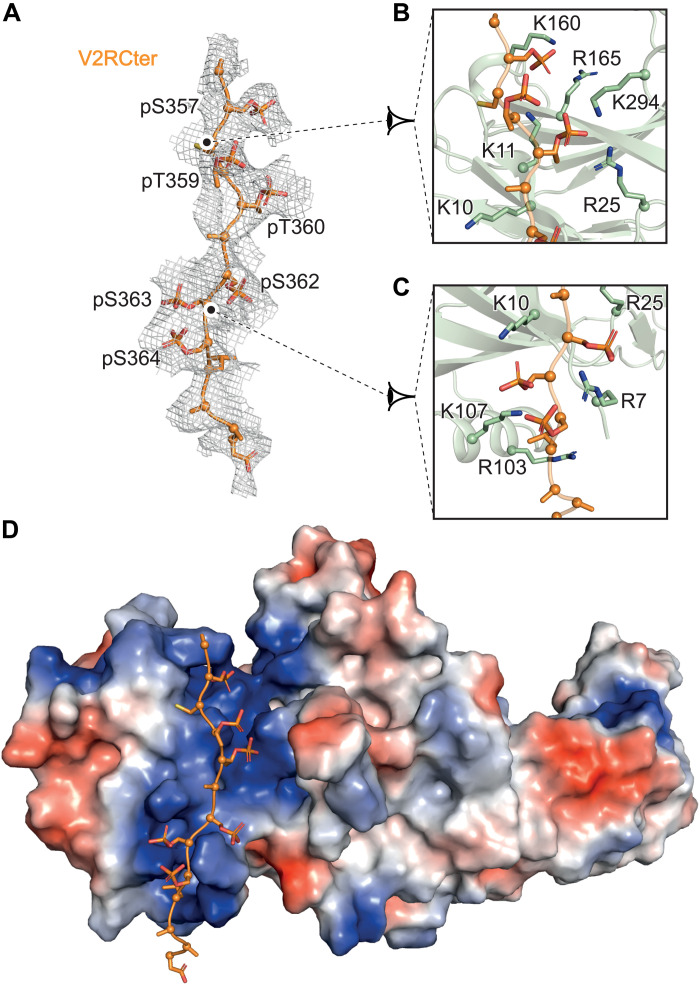
Phosphorylation of V2RCter and interaction with βarr1ΔCT N-lobe. (**A**) Overlay of the cryo-EM map of the V2RCter (mesh) with its corresponding model (in orange). Phosphate moieties of V2R pS357, pS359, pT369, pS362, pS363, and pS364 are shown in red. (**B** and **C**) Close-up views of these phosphorylated residues of the V2RCter and of positively charged residues (in pale green) of the N-lobe of βarr1ΔCT. (**D**) Global view of the phosphorylated V2RCter interacting with the N-lobe of βarr1ΔCT. The charge potential surface of βarr1ΔCT is shown in red (negatively charged residues) and blue (positively charged residues) representation.

Clear interactions between the Cter of V2R and the N-lobe of βarr1 are visualized in the density map of the locally refined βarr1ΔCT-ScFv30 subcomponents (Fig. 5A). This region of the complex displays better densities than elsewhere with a local resolution between 3.5 and 4 Å (fig. S7). Densities for V2R phosphoresidues S357, T359, T360, S362, S363, and S364 could be identified (Fig. 5A), in agreement with their position defined in the active structure of βarr1 in complex with a chemically synthesized V2Rpp (*18*) or with that seen in the M2R-βarr1 and β_1_AR-βarr1 complexes (both having a V2R Cter fused instead of their natural Cter). To confirm the presence of these phosphoresidues in the V2RCter, phosphoproteomics of the purified V2R has been performed using trypsin cleavage and the liquid chromatography–tandem mass spectrometry (LC-MS/MS) approach, allowing us to identify phosphopeptides and to determine the phosphosite localization (significant phosphosites are correlated to a localization probability superior to 0.75). Most of the phosphoresidues identified in the density map are indeed phosphorylated (fig. S15), since S357, T359, S362, and S364 present a localization probability above 0.75. In addition, T347 and S350, positioned right after helix 8, also display a high localization probability (but are not seen in the density map). Phosphoproteomic experiments also identified three phosphorylation sites in ICL3 (S241, T253, and S255, which are, however, not visible in the density map) (fig. S15), but we did not observe apparent interactions between these phosphorylated residues and βarr1ΔCT in MD simulations (table S2). Unexpectedly, a majority of the residues that are phosphorylated (both in the ICL3 and in the Cter) were posttranslationally modified whether the V2R-expressing Sf9 cells were stimulated or not with the full agonist AVP (1 μM for 30 min) before harvesting (fig. S15). This means that the V2R is constitutively phosphorylated in the Sf9 insect cells. The presence of active GRKs has been studied in this cellular system, and the role of these insect cell kinases has been proven in the agonist-induced desensitization and phosphorylation of the human M2 muscarinic and serotonin 5HT_1A_ receptors (*38*, *39*). In mammals, the V2R is physiologically expressed in principal cells of the collecting duct of the kidney nephron, which constitutes highly specialized polarized cells. By comparison, Sf9 cells that represent a recombinant overexpressing system may not recapitulate a complete pattern of endogenous V2R-associated signaling pathways and trafficking.

On the basis of the density map, the phosphoproteomic results, and previous structural data, we confidently built the interactions between negatively charged phosphates of the V2RCter residues and positively charged K/R residues of the βarr1 N-lobe (distances less than 4 Å) (Fig. 5, B to D). Phosphates of V2R S357, T359, and T360 are in close proximity to K11 and R25, whereas phosphates of V2R S362, S363, and S364 may establish ionic contacts with R7, K10, and K107 of βarr1 (Fig. 5, B and C), like in the crystal structure of the βarr1-V2Rpp-Fab30 (*18*) and in the structure of the M2R-βarr1 and β_1_AR-βarr1 complexes, where both GPCRs have a V2RCter fused instead of their natural sequence (*20*, *21*). The phosphorylation pattern of V2R we identified in the structure of the AVP-V2R-βarr1ΔCT-ScFv30 complex (and in the phosphoproteomic analysis) corresponds to the one in the crystal structure of the fully phosphorylated V2R in complex with βarr1 (*18*, *37*).

### Comparison of AVP-V2R-G_s_-Nb35 and AVP-V2R-βarr1ΔCT-ScFv30 complexes

**Fig. 6.**
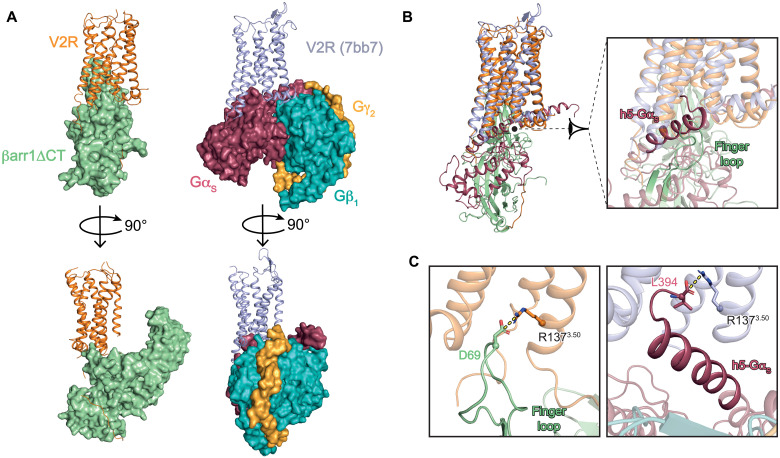
Comparison of AVP-V2R-G_s_ and AVP-V2R-βarr1ΔCT complexes. (**A**) Orthogonal views of V2R-βarr1ΔCT (left) and V2R-G_s_ (right, PDB 7bb7) complexes. The color scheme for the V2R and βarr1ΔCT is as in Fig. 1. In the other complex, V2R is shown in blue-gray, Gα_s_ in raspberry, Gβ_1_ in turquoise, and Gγ_2_ in yellow. (**B**) Superimposition of the two complexes with a close-up view at the interfaces. The C-terminal helix of the Gα subunit of the G_s_ protein (h5-Gα_s_ helix) and the tip of βarr1ΔCT FL insert into the TM core cavity of the V2R and overlap in the same binding space. (**C**) Overlapping of βarr1ΔCT FL and Gα_s_ Cter h5-helix binding sites in V2R. On the basis of what is described in M2R-βarr1 and β_1_AR-βarr1 complexes (*20*, *21*), the FL residue D69 carboxylic function may interact with V2R R137^3.50^ through an ionic bridge in the AVP-V2R-βarr1ΔCT complex. For comparison, the ionic interaction between the Cter free carboxylic function of the Gα_s_ with V2R R137^3.50^ in the AVP-V2R-G_s_ complex is shown.

We recently determined the structure of the AVP-bound V2R-G_s_ complex (*22*), which led us to compare it with the AVP-bound V2R-βarr1 complex (Fig. 6A). For both complexes, the interface between V2R and the signaling partners is native and was not modified by protein engineering. No mutations were introduced, no chimeric or fusion proteins were constructed, no cross-linking approach was done, and no NanoBIT tethering strategy was used (*40*). First, when comparing the structures of AVP-V2R-G_s_ (PDB 7bb7) and AVP-V2R-βarr1ΔCT complexes to the inactive structure of the related OTR (*33*), V2R TM6 moves outward in both cases with a similar range of distance, 13 Å (AVP-V2R-G_s_ assembly) versus 10 Å (AVP-V2R-βarr1ΔCT complex; Fig. 3B), leading to the opening of the receptor core cavity. Second, superposition of V2R from the two complexes (root mean square deviation of 3.5 Å for 1767 atoms) shows that both G_s_ and βarr1 insert into this core cavity (Fig. 6B). More precisely, the G_s_ protein α subunit Cter (h5 helix) and the tip of the βarr1 FL overlap in the same space (Fig. 6B). This has already been shown in previous studies. For instance, overlapping of the binding pockets of the visual arrestin FL and the transducin α subunit Cter helix into the TM bundle of active rhodopsin was demonstrated (*41*, *42*). Here, on the basis of the density maps and the corresponding models (Fig. 6, A and B), the main chain of D69 of the βarr1 FL would position its carboxylic acid function in a favorable position to form an ionic bond with R137^3.50^, as previously observed for the free carboxylic acid function of the G_s_ protein α subunit Cter (Fig. 6C) (*22*). The destabilization of the ionic lock motif is important for the receptor to reach active conformations and to engage with the different G protein– and βarr-dependent signaling pathways. Overall, the V2R conformation in the AVP-bound V2R-G_s_ complex is very similar to that in the AVP-bound V2R-βarr1ΔCT complex (Fig. 6B), but some key domains might present slight differences such as the TM7-H8 hinge. These key domains may be involved in biased ligand-induced differential conformations. For instance, using fluorescence spectroscopy and full versus biased ligands, the TM7-H8 hinge region was shown to favor βarr1 interaction over G protein (*43*).

Because each signaling protein is tightly associated with the V2R TM cavity in both assemblies, it is obvious that G_s_ and βarr1 cannot couple simultaneously when βarr1 is in the “core” conformation (Fig. 6A). These observations support a steric hindrance-based desensitization mechanism through competition for an overlapping interface at the cytoplasmic TM surface of the V2R (*44*, *45*). This is in agreement with studies showing that desensitization of G protein signaling (i.e., arresting the G protein signaling) is performed exclusively by the receptor core–engaged βarr1 (*29*, *46*), whereas a βarr1 in a “tail” conformation is fully capable of performing other canonical functions (i.e., signaling and receptor internalization). Simultaneous coupling of the two signaling proteins has also been demonstrated when arrestin is preassociated in the tail conformation, i.e., when the βarr1 only attaches to the phosphorylated Cter of the V2R (*44*). The formation of this megacomplex was proposed to provide a biophysical basis for sustained endosomal G protein signaling (*47*). Its structure has been solved recently (*45*). The AVP-V2R-βarr1ΔCT signaling complex described here only concerns the GPCR core–engaged βarr1 and might be representative of the desensitization step of G protein primary signaling regulated by the βarr1 at the plasma membrane.

The architecture of the AVP-V2R-βarr1ΔCT-ScFv30 complex described here notably puts forward an original positioning of the βarr1, an unusual V2R/βarr1 interface, and a strong tilt of βarr1ΔCT relative to V2R. This further highlights a notable structural variability among GPCR-arrestin signaling complexes. Although there are multiple sites of interactions between the V2R and βarr1ΔCT, as well as a structurally important interface between the micelle and the βarr1 C-edge, and despite the addition of stabilizing partners such as the ScFv30, this complex displays a highly flexible behavior. This is in agreement with multiple conformations of arrestins and their versatile role in biological signaling systems (*48*).

The AVP-V2R-βarr1 complex structure is an additional step to better understand receptor conformational changes upon binding to different signaling proteins. In the future, it would be crucial to determine how the structures of V2R-G_s_ and V2R-βarr1 complexes in the presence of biased ligands are conformationally different from those defined for unbiased AVP. Developing ligands able to discriminate the G_s_ protein– and βarr-dependent signaling pathways associated to V2R activation is of crucial importance regarding polycystic kidney disease (*49*) or two V2R-associated genetic diseases with opposite clinical outcomes, congenital nephrogenic diabetes insipidus (*50*) and nephrogenic syndrome of inappropriate antidiuresis (*51*). Structure-based development of novel molecules able to differentiate G_s_ protein–, core βarr1–, or tail βarr1–associated V2R conformations will pave the way to design better drugs against kidney pathologies (*52*).

## MATERIALS AND METHODS

### Data analysis and figure preparation

Figures were created using the PyMOL 2.3.5 Molecular Graphics System (Schrödinger LLC) and the UCSF Chimera X 0.9 package (*53*). Data were plotted with GraphPad Prism 9.1.1 (GraphPad Prism Software Inc.).

### V2R expression and purification

The optimized sequence of the human V2R was cloned into the pFastBac1 vector (Thermo Fisher Scientific) using Eco RI/Xba I restriction sites to enable insect Sf9 cell infection using a baculovirus cell expression system. Since it has been demonstrated that the whole C terminus of V2R is crucial for arrestin interaction (*13*), it was conserved native in our construct. The hemagglutinin signal peptide (MKTIIALSYIFCLVFA), a first Flag-tag (DYKDDDDA), a Twin-Strep-tag (WSHPQFEKGGGSGGGSGGGSWSHPQFEK), a human rhinovirus 3C (HRV3C) protease cleavage site, and a second Flag-tag were all inserted in frame in the V2R N terminus to facilitate the expression and purification of the receptor (fig. S1A). N22 was substituted with a glutamine residue to avoid *N*-glycosylation. M1 and L2 residues of the wild-type V2R sequence were not present in this construct. Before production in Sf9 insect cells, this construct was first validated in human embryonic kidney (HEK) cells to control that it retained wild-type pharmacological and functional properties. First, the dissociation constant (*K*_d_) of the fluorescently labeled antagonist was 4.22 ± 0.7 nM (*n* = 3), in agreement with that defined for the wild-type V2R (*54*). Then, AVP binding, accumulation of cytosolic cAMP, and recruitment of βarr2 assays (see below for description of the three methods) all confirmed that the V2R is fully functional (fig. S2). The inhibition constant (*K*_i_) for AVP was 3.17 ± 0.97 nM (*n* = 3), and the activation constant (*K*_act_) for AVP-induced cAMP production and arrestin recruitment was 0.22 ± 0.09 nM (*n* = 3) and 2.02 ± 0.28 nM (*n* = 4), respectively, in accordance with those determined for a wild-type V2R (*22*, *54*, *55*).

The V2R was expressed in Sf9 insect cells using the Bac-to-Bac baculovirus expression system (Thermo Fisher Scientific) according to the manufacturer’s instructions, as previously described (*22*). Briefly, insect cells were grown in suspension in EX-CELL 420 medium (Sigma-Aldrich) to a density of 4 × 10^6^ cells/ml and infected with the recombinant baculovirus at a multiplicity of infection of 2 to 3. The culture medium was supplemented with the V2R pharmacochaperone antagonist tolvaptan (TVP) (Sigma-Aldrich) at 1 μM to increase the receptor expression levels (*56*, *57*). The cells were infected for 48 to 54 hours at 28°C, and expression of the V2R was checked by immunofluorescence using an anti-Flag M1 antibody coupled to Alexa Fluor 488. On the basis of the literature where it is described that V2R phosphorylation is agonist dependent, at least in mammalian cell systems such as transfected HEK or COS cells (*58*), cells were treated with 1 μM AVP 30 min before being harvested by centrifugation (two steps for 20 min at 3000*g*), and pellets were stored at −80°C until use.

The first step of V2R purification was performed as already described (*22*). Briefly, the cell pellets were thawed and lysed by osmotic shock in 10 mM tris-HCl (pH 8), 1 mM EDTA buffer containing iodoacetamide (2 mg/ml; Sigma-Aldrich), 1 μM TVP, and protease inhibitors [leupeptine (5 μg/ml) (Euromedex), benzamidine (10 μg/ml) (Sigma-Aldrich), and phenylmethylsulfonyl fluoride (PMSF) (10 μg/ml) (Euromedex)]. After centrifugation (15 min at 38,400*g*), the pellet containing crude membranes was solubilized using a glass Dounce tissue grinder (15 and 20 strokes using A and B pestles, respectively) in a solubilization buffer containing 20 mM tris-HCl (pH 8), 500 mM NaCl, 0.5% (w/v) *n*-dodecyl-β-d-maltopyranoside (DDM; Anatrace), 0.2% (w/v) sodium cholate (Sigma-Aldrich), 0.03% (w/v) CHS (Sigma-Aldrich), 20% glycerol, iodoacetamide (2 mg/ml), biotin BioLock (0.75 ml/liter, IBA), 1 μM TVP, and protease inhibitors. The extraction mixture was stirred for 1 hour at 4°C and centrifuged (20 min at 38,400*g*). The cleared supernatant was poured onto an equilibrated Strep-Tactin resin (IBA) for a first affinity purification step. After 2 hours of incubation at 4°C under stirring, the resin was washed three times with 10 column volumes (CV) of a buffer containing 20 mM tris-HCl (pH 8), 500 mM NaCl, 0.1% (w/v) DDM, 0.02% (w/v) sodium cholate, 0.03% (w/v) CHS, and 1 μM TVP. The bound receptor was eluted in the same buffer supplemented with 2.5 mM desthiobiotin (IBA). The HRV3C protease was added for overnight cleavage at 4°C [a 1:20 (HRV3C:V2R) weight ratio]. After digestion, the eluate was loaded onto an M2 anti-Flag affinity resin (Sigma-Aldrich). After loading, the DDM detergent was then gradually exchanged with LMNG (Anatrace) and GDN (Anatrace). The LMNG concentration was then decreased gradually from 0.5 to 0.02% and that of GDN decreased from 0.125 to 0.005%. The V2R was eluted in 20 mM Hepes (pH 7.5), 100 mM NaCl, 0.02% LMNG, 0.005% GDN, 0.002% CHS, 10 μM AVP (Bachem), and Flag peptide (0.4 mg/ml; Covalab). After concentration using a 50-kDa molecular weight cutoff (MWCO) concentrator (Millipore), the V2R was purified by size exclusion chromatography (SEC) using a Superdex 200 Increase (10/300 GL column) connected to an ÄKTA purifier system (GE Healthcare). Fractions corresponding to the pure monomeric receptor were pooled (~2 ml) and concentrated to 50 to 100 μM with an excess of AVP (200 μM).

### βarr1 expression and purification

A truncated version of βarr1 at residue 382 (βarr1ΔCT) was produced and purified (fig. S1B), since it has been shown to display a constitutive activity in cells (*59*). This βarr1 variant was able to effectively desensitize β_2_AR and δ opioid receptor in *Xenopus* oocytes. In addition, its recombinantly purified version was demonstrated to stably interact with the purified NTSR1 (*27*). It was prepared as follows. BL21(DE3) competent *Escherichia coli* cells (Thermo Fisher Scientific) were transformed using a pET plasmid containing an optimized version of β-arr1ΔCT fused to a Twin-Strep-tag sequence at its N terminus (Nco I/Xho I subcloning), and large-scale cultures were grown in LB + kanamycin at 37°C (170 rpm) until an optical density at 600 nm at 0.6 U was reached. Cells were induced at 37°C for 5 hours by adding 0.025 mM isopropyl-β-d-thiogalactopyranoside. Cells were collected by centrifugation (two steps for 20 min at 3000*g*), and pellets were stored at −80°C until use. Cells were resuspended in lysis buffer [20 mM tris-HCl (pH 8), 1 mM EDTA, 200 mM NaCl, and 1 mM β-mercaptoethanol] supplemented with protease inhibitors [leupeptine (5 μg/ml), benzamidine (10 μg/ml), and PMSF (10 μg/ml)]. Cells were lysed by sonication, and the lysate was supplemented with MgCl_2_ (5 mM final) and Benzonase (2000 U). After centrifugation (20 min, 4°C, 38,400*g*), the supernatant was supplemented with biotin BioLock (0.75 ml/liter) and loaded to Strep-Tactin affinity resin at 4°C. The resin was washed with 20 CV of wash buffer [20 mM tris (pH 8), 200 mM NaCl, and 100 μM Tris(2-carboxyethyl)phosphine (TCEP)]. The protein was then eluted with 5 CV of wash buffer supplemented with 2.5 mM desthiobiotin (IBA). Subsequently, it was subjected to a Superdex 200 Increase gel filtration step (10/300 GL column) with a buffer containing 20 mM Hepes (pH 7.5), 200 mM NaCl, and 100 μM TCEP. The fractions corresponding to the purified βarr1ΔCT were collected, concentrated to approximately 250 μM using a 10-kDa MWCO concentrator (Millipore). Aliquots were then flash-frozen and stored at −80°C until use.

### ScFv30 expression and purification

The single-chain variable fragment ScFv30 with a Twin-Strep-tag added at its C terminus was used in this study to enable a stable interaction between the receptor and βarr1ΔCT (*18*, *60*). Moreover, this antibody fragment has been shown to not bind to the C terminus of βarr1, and the corresponding Fab30 was shown to lock the βarr1 in its active conformation (*21*). Briefly, the optimized nucleotide sequence of ScFv30 was cloned in Nco I/Pme I restriction sites of a modified pMT/BIP/V5 vector (Life Technologies) in which the V5 epitope and the 6-His tag were replaced by an enterokinase cleavage site followed by a Twin-Strep-tag (WSHPQFEKGGGSGGGSGGGSWSHPQFEK). This vector is adapted to the secreted expression of recombinant proteins in *Drosophila melanogaster* S2 Schneider cell cultures (*61*, *62*). In this plasmid, the ScFv30 sequence is in frame with the BIP signal sequence and the Twin-Strep-tag. The expression and purification of ScFv30 were performed as follows. S2 Schneider cells (Life Technologies), cultured in serum-free insect Xpress medium (Lonza), were transfected as reported previously (*63*) and amplified, and ScFv30 expression was induced with 4 μM CdCl_2_ at a density of ~10 × 10^6^ cells/ml for 6 to 8 days for large-scale production.

Cells were harvested by centrifugation (38,400*g*, 10 min, 4°C) to remove cells and cellular debris. Then, protease inhibitors [leupeptine (5 μg/ml), benzamidine (10 μg/ml), and PMSF (10 μg/ml)] were added to the supernatant. The sample was filtered and concentrated at 4°C using a Vivaflow 200 cassette with a 10-kDa MWCO (Sartorius). When the volume was reaching around 100 ml, tris-HCl (pH 8; 100 mM) and 0.5 ml of BioLock (IBA) were added. The ScFv30 was purified by Strep-Tactin affinity chromatography (IBA) in a buffer containing 100 mM tris-HCl (pH 8), 150 mM NaCl, and 1 mM EDTA. The eluate was concentrated to reach a 5- to 10-ml volume and dialyzed in two steps (overnight at 4°C, then 2 hours) in a buffer containing 20 mM Hepes (pH 7.5) and 100 mM NaCl. The dialyzed ScFv30 was concentrated to approximately 200 μM using a 10-kDa MWCO concentrator (Millipore). Aliquots were flash-frozen in liquid nitrogen and stored at −80°C until use.

### Purification of the AVP-V2R-βarr1ΔCT-ScFv30 complex

As indicated above, all components of the complex (V2R, βarr1ΔCT, and ScFv30) were first expressed and purified separately. Then, they were mixed in the presence of an excess of AVP (fig. S3). The mixture was complemented with a PtdIns(4,5)P_2_ analog, the dioctyl-PtdIns(4,5)P_2_ (diC8PIP2), for two main reasons: (i) An inositol phosphate binding site was described at the top of the C-lobe of both βarr1 and βarr2 (*32*, *64*), and (ii) the same analog diC8PIP2 was shown to be important for NTSR1-βarr1 complex formation (*27*).

Briefly, V2R was mixed with an equimolar concentration of diC8PIP2 (Avanti Polar Lipids Inc.), an excess of βarr1ΔCT (2:1 molar ratio), and an excess of ScFv30 (2:1 molar ratio) as well as 250 μM AVP and 2.5 mM MgCl_2_. In a representative experiment, concentrations of the different components of the complex were as follows: 35 μM V2R and diC8PIP_2_ and 70 μM βarr1ΔCT and ScFv30. The coupling reaction was allowed to proceed at room temperature (RT) for 90 min. The complex was then purified through an orthogonal affinity chromatography procedure followed by an SEC step. First, to remove excess of βarr1ΔCT and ScFv30, the complex AVP-V2R-βarr1ΔCT-ScFv30 was purified by an M2 anti-Flag affinity chromatography. The mixture was loaded three times on the column, and the resin was washed three times with 10 CV of wash buffer containing 20 mM Hepes (pH 7.5), 100 mM NaCl, 0.002% CHS, 0.02% LMNG, 0.005% GDN, and 10 μM AVP. The complex and the uncomplexed V2R were then eluted with 5 CV of wash buffer supplemented with Flag peptide (400 μg/ml). Second, the eluate was then loaded onto Strep-Tactin affinity resin to get rid of the uncomplexed receptors. The resin was washed with 10 CV of wash buffer [20 mM Hepes (pH 7.5), 100 mM NaCl, 0.02% LMNG, 0.005% GDN, 0.002% CHS, and 10 μM AVP]. The complex was then eluted with 5 CV of wash buffer complemented with 2.5 mM desthiobiotin. Last, the eluate was concentrated with a 50-kDa MWCO concentrator and subjected to an SEC Superose 6 (10/300 GL, GE Healthcare) equilibrated with a buffer containing 20 mM Hepes (pH 7.5), 100 mM NaCl, 0.0011% LMNG, 0.001% GDN, 0.002% CHS, and 10 μM AVP. The complex displayed a monodisperse peak whose analysis by SDS polyacrylamide gel and Coomassie blue staining confirmed the presence of all proteins (fig. S3). Peak fractions were pooled, supplemented with 0.001% amphipol A8-35, and concentrated using a 50-kDa MWCO concentrator to ~3 mg/ml for cryo-EM studies.

### Negative stain microscopy observations

Before preparing cryo-EM grids, we first checked the quality and the homogeneity of the AVP-V2R-βarr1ΔCT-ScFv30 samples by negative stain EM (NS-EM). Three microliters of each complex at 0.04 mg/ml was applied for 2 min on glow-discharged carbon-coated grids and then negatively stained with 0.75% uranyl formate for 1 min. Observation of EM grids was carried out on a JEOL 2200FS FEG Transmission Electron Microscope (TEM) operating at 200 kV under low-dose conditions (total dose of 20 electrons/Å^2^) in the zero–energy loss mode with a slit width of 20 eV. Images were recorded on a 4K × 4K slow-scan charge-coupled device camera (Gatan Inc.) at a nominal magnification of ×50,000 with defocus ranging from 0.5 to 1.5 μm. In total, 55 micrographs were recorded, allowing us to pick 97,182 particles using e2boxer from Eman2 package (*65*). Further processing was performed with Relion 3.1. The particles were subjected to a 2D classification, including to get rid of free micelles and dissociated components of the complex. From 2D classes, 65,090 particles corresponding to the AVP-V2R-βarr1ΔCT-ScFv30 complexes were selected, representing 67% of all particles picked, a good prerequisite for cryo-EM analysis. The AVP-V2R-βarr1ΔCT-ScFv30 complex revealed a homogeneous distribution of particles showing a two-domain organization (V2R in the detergent micelle versus βarr1ΔCT-ScFv30). The 2D class averages clearly showed the βarr1ΔCT tightly engaged within the micelle-embedded V2R (fig. S3), an architecture in agreement with the core conformation of the GPCR-βarr1 complex (*29*).

### Cryo-EM sample preparation and image acquisition

For AVP-V2R-βarr1ΔCT-ScFv30 cryo-EM investigation, 3-μl samples were applied on glow-discharged Quantifoil R1.2/1.3 300-mesh UltrAufoil grids (Quantifoil Micro Tools GmbH, Germany), blotted for 3.5 s, and then flash-frozen in liquid ethane using the semiautomated EM GP2 (Leica Microsystems) plunge freezer (100% humidity and 4°C). Images were collected in one session at the European Molecular Biology Laboratory (EMBL) of Heidelberg (Germany) on a FEI Titan Krios (Thermo Fisher Scientific) at 300 keV through a Gatan Quantum 967 LS energy filter using a 20-eV slit width in zero-loss mode and equipped with a K3 Summit (Gatan Inc.) direct electron detector configured in counting mode. Movies were recorded at a nominal energy-filtered TEM magnification of ×130,000 corresponding to a 0.64-Å calibrated pixel size. The movies were collected in 40 frames in defocus range between −1 and −2 μm with a total dose of 52.63 e^−^/Å^2^. Data collection was fully automated using SerialEM, resulting in 14,080 movies.

### Cryo-EM data processing

Movie frames were aligned and summed using the MotionCorr Relion own implementation (Relion 3.1.2) with seven-by-five patches, a *B* factor of 150, and a binning factor of 2, resulting in motion-corrected images with a pixel size of 1.28 Å. The contrast transfer function parameters were estimated using Gctf (*66*). The images with a maximal resolution estimation worse than 7 Å were discarded, resulting in 13,566 images (fig. S4A). A first automatic picking (fig. S5) was carried out using boxnet from Warp software package (*67*), allowing us to select and extract 3,610,370 particles, which were transferred into Relion v3.1.2. Iterative 2D classifications combined with bad 2D class average exclusion sorted out a total of 1,169,437 particles (fig. S4A). Those particle coordinates were used as references to train a model with Topaz (*68*), a positive-unlabeled convolutional neural network for particle picking (fig. S5). It resulted in the picking of 4,595,394 particles. Both datasets of particles were transferred into Relion 3.1.2 and subjected separately to iterative 2D classifications. The particles selected from the best 2D class averages (1,806,545 particles from boxnet and 2,660,410 particles from Topaz) were then merged and duplicates were removed, yielding a dataset of 3,721,020 particles. A conventional approach (fig. S4B) was first performed with two rounds of 2D classification, allowing us to select 2,206,913 particles, and subjected to an initial model reconstruction and diverse iterative 3D classifications. Although playing with multiple different parameters (*T* value, mask size, number of 3D classes, box size, and Relion vs cryoSPARC), this approach only allowed us to compute a density map at 6.3 Å resolution. An optimized approach (fig. S4C) was then used with first iterative 2D classifications in Relion, based on particle orientation through the use of different mask size, yielding a total of 729,335 particles from best 2D class averages. Several rounds of cryoSPARC v3.2.0 were then performed using 2D classification followed by iterative ab initio reconstruction processing steps (six times using two models), resulting in the selection of a particle stack comprising 27,637 particles that yielded a density map with an overall resolution of 4.75 Å after 3D NU refinement (figs. S4C and S6). Resolution was estimated using the gold standard FSC at 0.143 (FSC = 0.143). This stack of particles was transferred into Relion for micelle-V2R signal subtraction. The particle boxes were recentered and adapted to the size of the βarr1-ScFv30 complex. Subtracted particles were subjected to local refinement in cryoSPARC, yielding a density map (EMD-14223) with an overall resolution of 4.23 Å (FSC = 0.143) (fig. S7). Attempts to align the complex with micelle subtraction yielded density maps with similar apparent quality and overall resolution than without subtraction (*r* ~ 4.8 to 5 Å). Attempts to align the V2R alone were unsuccessful. The subset of 27,637 particles was then refined using additional ab initio steps to obtain a subset of 8296 particles, which yielded a density map (EMD-14221) with an overall resolution of 4.73 Å after NU refinement (fig. S7). This map displays more detailed features of the dynamic regions of the complex as compared to the initial 27,637-particle density map. Local resolution of the two maps calculated using cryoSPARC (EMD-14221 and EMD-14223) ranged from 3.5 to 5.5 Å. The *E*_od_, a coefficient related to the orientation distribution via its corresponding point spread function (*69*), calculated from the 4.23-Å- and 4.73-Å-resolution maps, was substantially above the 0.6 threshold (0.88 and 0.8, respectively), indicating a uniform resolution of maps in all directions, although some orientations have been discarded upon the process. During the optimized approach, multiple 3D variability analyses were also performed using cryoDRGN, cryoSPARC (movie S1), and Relion with different quantities of particles. Unfortunately, none of the methods brought exploitable data.

### Model building and refinement

For each of the two cryo-EM maps [EMD-14221 (full complex) and EMD-14223 (focused around the βarr1ΔCT-ScFv30 substructure)], an atomic model was built and refined. A starting model of V2RCter-βarr1ΔCT-ScFv30 substructure was built using the atomic structure of the V2RCter (residues 356 to 368 according to residue numbering of UniProt entry P30518) from PDB 6u1n (4-Å resolution) and the Fab30 and βarr1 from PDB 4jqi (2.6-Å resolution). The model was fitted into the EMD-14223 map and manually adjusted (sequence mutations and loop reconstruction) using Coot (*70*). Then, this intermediate model was rigid body–fitted into the EMD-14221 map and completed with the structure of the V2R TM domain + AVP from PDB 7kh0 (2.8-Å resolution) and a molecule of diC8PIP2. The resulting model of the full complex (AVP-V2R-βarr1ΔCT-ScFv30) was real space–refined against the EMD-14221 map under standard stereochemical restraints (including Ramachandran restraints) using Coot (*70*) and Phenix (*71*). The refined model of the full complex (PDB 7Rr0c) does not include the V2R N-terminal portion (residues 1 to 31), parts of the V2R ICLs (residues 148 to 156 from ICL1, residues 183 to 188 in ICL2, and residues 239 to 263 in ICL3), and parts of the V2R Cter (residues 343 to 355 and 369 to 371), which were not visible in the density maps. The model of βarr1ΔCT in PDB 7r0c includes residues 6 to 365 (except residues 332 to 339), whereas ScFv30 is nearly complete (residues 110 to 128 are missing). The intermediate model of the V2RCter-βarr1ΔCT-ScFv30 substructure (initially fitted in the EMD-14223 map) was further real space–refined (with Phenix and Coot) against the EMD-14223 map by temporarily including the V2R TM domain from PDB 7r0c (to avoid βarr1ΔCT residues jumping into V2R residual density still present in proximity of βarr1ΔCT) and by imposing extremely strong geometrical constraints on the V2R model (to avoid unjustified modifications of the V2R regions farther from the βarr1ΔCT where no density was left after the signal subtraction procedure). The V2R TM domain was lastly removed from the refined V2RCter-βarr1ΔCT-ScFv30 model (PDB 7r0j).

We have also applied a combination of MD approaches (using Chimera X/ISOLDE with the MDFF option checked) that was validated using Coot and Phenix. The obtained model with MD does not substantially improve the model, showing a difference only on the C-edge loop, a dynamic area that is not represented in the initial model. This is not unexpected because of the lack of density map and of the dynamic in this area. For those reasons, the initial model was thus conserved for PDB deposition.

### LC-MS/MS and analysis of V2R phosphoresidues

To prepare samples for LC-MS/MS, purification of the V2R was adapted from a previously described protocol (see above in the “V2R expression and purification” section) (*22*). As indicated, the receptor was first purified by affinity chromatography using Strep-Tactin resin (IBA) following the same protocol. Then, the eluate was then loaded onto an M2 anti-Flag affinity resin (Sigma-Aldrich). The column was washed first with 10 CV of a buffer containing 20 mM Hepes (pH 7.5), 100 mM NaCl, 0.1% (w/v) DDM, 0.01% (w/v) CHS, and 10 μM TVP. A second wash followed with 10 CV of a buffer containing 20 mM Hepes (pH 7.5), 100 mM NaCl, 0.025% (w/v) DDM, 0.005% (w/v) CHS, and 10 μM TVP. The bound receptor was eluted in the same buffer supplemented with FLAG peptide (0.4 mg/ml) (5 CV). The fractions corresponding to the receptor were collected, and the HRV3C protease was added for overnight cleavage at 4°C [at a 1:20 (HRV3C:V2R) weight ratio]. After concentration using a 50-kDa MWCO concentrator (Millipore), the V2R was purified by SEC using a Superdex 200 Increase (10/300 GL column) connected to an ÄKTA purifier system (GE Healthcare). Fractions corresponding to the pure monomeric receptor were pooled (~1.5 ml) and concentrated to 30 μM (1.2 mg/ml).

The purified V2R (100 to 200 μg) was digested using micro S-Trap columns (https://protifi.com/; Huntington, NY) following the supplier’s protocol. Briefly, after reduction (20 mM dithiothreitol for 10 min at 95°C) and alkylation (40 mM indole-3-acetic acid for 30 min in the dark), the receptor was digested using 3 μg of trypsin (Promega, Gold) for 1 hour at 47°C. The peptides obtained were analyzed using nano-throughput high-performance liquid chromatography (Ultimate 3000-RSLC, Thermo Fisher Scientific) coupled to a mass spectrometer (Q Exactive-HF, Thermo Fisher Scientific) equipped with a nanospray source. The preconcentration of the samples was carried out in line on a precolumn (0.3 mm × 10 mm, Pepmap, Thermo Fisher Scientific) and separation of the peptides was carried out on a column (0.075 mm × 500 mm, reverse phase C18, Pepmap, Dionex) following a gradient from 2 to 25% buffer B [0.1% Formic acid (AF) in 80% acetonitrile (ACN)] for 100 min at a flow rate of 300 nl/min, then 25 to 40% in 20 min, and, lastly, 40 to 90% in 3 min.

The spectra were acquired in “data-dependent acquisition” (dynamic exclusion of 20 s) mode. The LC-MS/MS analysis cycle is therefore composed of several phases, a “full scan MS” with analysis in the orbitrap at 60,000 resolution followed by MS/MS [higher-energy collisional dissociation (HCD) fragmentation], for the 12 most abundant precursors at a resolution of 30,000. Raw spectra were processed using the MaxQuant environment v1.6.10.43 (*72*) and Andromeda for database search with match between runs and the iBAQ algorithm enabled. The MS/MS spectra were matched against the sequence of the V2R construct (fig. S15), the reference proteome (Proteome ID UP000008292, release 2021_01) of *Autographa californica* nuclear polyhedrosis virus, the UniProt entries (release 2021_01, www.uniprot.org/) for *Spodoptera frugiperda* (fall armyworm, taxon identifier 7108), and 250 frequently observed contaminants, as well as reversed sequences of all entries. Enzyme specificity was set to trypsin/P, and the search included cysteine carbamidomethylation as a fixed modification and oxidation of methionine, acetylation (protein N terminus), and phosphorylation of Ser, Thr, and Tyr residues (STY) as variable modifications. Up to two missed cleavages were allowed for protease digestion. The maximum false discovery rate for peptides and proteins was set to 0.01. Representative protein ID in each protein group was automatically selected using the in-house developed Leading tool v3.4 (*73*). Signal intensities of receptor peptides were extracted using Skyline v2.1.0.31 (*74*), with the option “use high-selectivity extraction.” For a defined peptide sequence, a score corresponding to the probability of phosphorylation for each possible position (S, T, or Y) was determined (fig. S15). The normalized sum of all these probabilities is then used to define the confidence of localization, known as localization probability (*75*). Classically, class I phosphosites correspond to sites with a localization probability of at least 0.75 (*76*). Residues identified as phosphorylated in the density map (such as T360 or S363) may, however, present a localization probability under 0.75 using the phosphoproteomic approach. This apparent discrepancy can be explained. For instance, cleavage of the V2R generates the peptide R_346_TPPSLGPQDESCTTASSSLAKD_368_, which corresponds in part to the C-terminal portion of the receptor. This peptide contains eight potential phosphorylation sites. Such a quantity of T and S residues, in addition to their proximity, makes it difficult to precisely locate all the phosphorylation moieties, and we can only propose a probability score of the presence or absence of a phosphate group for a specific amino acid. Even if a threshold of 0.75 has been chosen as significative, scores of 0.62 (T360) or 0.66 (S363) do not exclude the possibility of phosphorylation at these positions. Also, we have to consider that the detection of the phosphorylation was made on a sample containing multiple proteins including different phosphorylation patterns. Knowing that, and because our cryo-EM maps represent only a subset of particles (27,637 particles following 2D and 3D classification) corresponding to the most representative population allowing to reach high resolution, it is thus not unexpected that we have mostly selected a population where the C-terminal portion of the V2R in contact with the βarr1 is fully phosphorylated. Such a phosphorylation profile is, for example, in agreement with the one seen in the muscarinic M2R–arrestin2 complex using a V2Rpp peptide (containing the same eight phosphorylation sites observed in our map) (*20*) or with the fully activated V2R C-terminal peptide in complex with the arrestin (*18*, *37*).

### Time-resolved fluorescence resonance energy transfer binding assays

V2R binding studies using Tag-lite assays (PerkinElmer Cisbio) based on time-resolved fluorescence resonance energy transfer (TR-FRET) measurements were previously described (*22*, *54*, *77*). Briefly, HEK cells were plated in white-walled, flat-bottom, 96-well plates (Greiner CELLSTAR plate, Sigma-Aldrich) in Dulbecco’s minimum essential medium (DMEM) containing 10% fetal bovine serum (Eurobio), 1% nonessential amino acids (GIBCO), and penicillin/streptomycin (GIBCO) at 15,000 cells per well. Cells were transfected 24 hours later with a plasmid coding for the V2R version used in cryo-EM studies fused at its N terminus to the SNAP-tag (SNAP-V2R) (PerkinElmer Cisbio). Transfections were performed with X-tremeGENE 360 (Merck), according to the manufacturer’s recommendations: 10 μl of a premix containing DMEM, X-tremeGENE 360 (0.3 μl per well), SNAP-V2 coding plasmid (30 ng per well), and noncoding plasmid (70 ng per well) was added to the culture medium. After a 48-hour culture period, cells were rinsed once with Tag-lite medium (PerkinElmer Cisbio) and incubated in the presence of Tag-lite medium containing 100 nM benzylguanine-Lumi4-Tb for at least 60 min at 37°C. Cells were then washed four times. For saturation studies, cells were incubated for at least 4 hours at 4°C in the presence of a benzazepine-red nonpeptide vasopressin antagonist (BZ-DY647, PerkinElmer Cisbio) at various concentrations ranging from 1 × 10^−10^ to 1 × 10^−7^ M. Nonspecific binding was determined in the presence of 10 μM vasopressin. For competition studies, cells were incubated for at least 4 hours at 4°C with benzazepine-red ligand (5 nM) and increasing concentrations of vasopressin ranging from 1 × 10^−11^ to 3.16 × 10^−6^ M. Fluorescent signals were measured at 620 nm (fluorescence of the donor) and at 665 nm (FRET signal) on a PHERAstar (BMG LABTECH). Results were expressed as the 665/620 ratio [10,000 × (665/620)]. A specific variation of the FRET ratio was plotted as a function of benzazepine-red concentration (saturation experiments) or competitor concentration (competition experiment). All binding data were analyzed with GraphPad 9.1.1 (GraphPad Prism Software Inc.) using the one site-specific binding equation. All results are expressed as the means ± SEM of at least three independent experiments performed in triplicate (fig. S2). *K*_i_ values were calculated from median inhibitory concentration values with the Cheng-Prusoff equation.

### cAMP accumulation assays

V2R functional studies based on TR-FRET measurements were described previously (*22*, *57*, *78*). Briefly, HEK cells were plated in black-walled 96-well plates (Falcon) at 15,000 cells per well. Cells were transfected 24 hours later with a plasmid coding for the V2R version used in cryo-EM studies. Transfections were performed with X-tremeGENE 360 (Merck), according to the manufacturer’s recommendations: 10 μl of a premix containing DMEM, X-tremeGENE 360 (0.3 μl per well), SNAP-V2 coding plasmid (0.002 ng per well), and noncoding plasmid (100 ng per well) was added to the culture medium. After a 24-hour culture period, cells were treated for 30 min at 37°C in the cAMP buffer with or without increasing AVP concentrations (3.16 × 10^−12^ to 10^−6^ M) in the presence of 0.1 mM RO201724, a phosphodiesterase inhibitor (Sigma-Aldrich). The accumulated cAMP was quantified using the cAMP Dynamic 2 Kit (PerkinElmer Cisbio) according to the manufacturer’s protocol. Fluorescent signals were measured at 620 and 665 nm on a Spark 20M multimode microplate reader (Tecan). Data were plotted as the FRET ratio [10,000 × (665/620)] as a function of AVP concentration [log (AVP)]. Data were analyzed with GraphPad Prism v9.1.1 (GraphPad Prism Software Inc.) using the “dose-response stimulation” subroutine. Median effective concentrations were determined using the log(agonist) versus response variable slope (four-parameter) fit procedure. Experiments were repeated at least three times on different cultures, with each condition in triplicate. Data are presented as means ± SEM (fig. S2).

### β-Arrestin recruitment assays

Upon GPCR activation, β-arrestins (βarr) are recruited to stop G protein signaling and to initiate clathrin-mediated receptor internalization. During this process, the release of the C-terminal domain of βarrs is associated with the binding of βarrs to the adaptor protein 2 (AP2). This interaction can be measured using the HTRF technology (PerkinElmer CisBio) based on the use of two specific antibodies, one directed against βarr2, and the second one specific for AP2. In this assay (βarr2 recruitment kit, PerkinElmer CisBio), the AP2 antibody is labeled with a Europium (Eu) cryptate fluorescent donor, and the one against βarr2 is labeled with a d2 fluorescent acceptor, with their proximity being detected by FRET signals. The specific signal is positively modulated in proportion with the recruitment of βarr2 to AP2 upon V2R activation by AVP. Briefly, HEK cells were plated at a seeding density of 2.5 × 10^4^ cells per well in white-walled 96-well plates (CELLSTAR plate, Sigma-Aldrich) precoated with poly-l-ornithine (14 μg/ml; Sigma-Aldrich) for 24 hours, in DMEM (GIBCO) complemented with 10% fetal bovine serum (Eurobio), 1% nonessential amino acids (GIBCO), and 1% penicillin-streptomycin antibiotic solution (GIBCO). To produce the V2R, the cells were transfected with 30 ng of the pRK5-Flag-Snap-V2R plasmid (coding for the cleaved V2R construct used in cryo-EM studies) using X-tremeGENE 360 (Merck), according to the manufacturer’s recommendations. After a 24-hour culture, cells were used to evaluate the recruitment of βarr2 to AP2 upon V2R activation with the βarr2 recruitment kit (PerkinElmer CisBio), following the manufacturer’s recommendation. Briefly, the cells were first washed once with DMEM and incubated for 2 hours at RT with 100 μl per well of stimulation buffer containing various concentrations of the ligand AVP (ranging from 10^−6^ to 10^−12^ M). The medium was then replaced by 30 μl per well of stabilization buffer for 15 min at RT. The cells were then washed three times with 100 μl per well of wash buffer before adding 100 μl per well of a premix of Eu cryptate and d2 antibodies in detection buffer. Following overnight incubation at RT, 80 μl of medium was removed from each well before reading the 96-well plates on a Spark 20M multimode microplate reader (Tecan) or a PHERAstar FS (BMG Labtech) by measuring the signals of the donor (Europium cryptate–labeled AP2 antibody) at a wavelength of 620 nm and of the acceptor at 665 nm (d2-labeled βarr2). Last, the results were expressed as the FRET ratio [(665/620) × 10,000] and plotted using GraphPad 9.1.1 (GraphPad Prism Software Inc.). Experiments were repeated at least three times on different cultures, with each condition in triplicate. Data are presented as means ± SEM (fig. S2).

### MD simulation method

MD simulations were performed for the AVP-V2R-βarr1ΔCT complex with and without diC8PIP2 (figs. S10 and S11). The initial models were built from the cryo-EM structure reported here. The missing ICL2 was modeled on the basis of the cryo-EM structure of AVP-V2R-G_s_ (PDB 7dw9) (*24*). Other missing loop regions were generated by Modeller v9.15 (*79*). Residues S241^ICL3^, T253^ICL3^, S255^ICL3^, S357^Cter^, T359^Cter^, T360^Cter^, S362^Cter^, S363^Cter^, and S364^Cter^ of V2R were phosphorylated. PACKMOL-Memgen (*80*) was used to assign the side-chain protonation states and embed the models in a bilayer of 1-palmitoyl-2-oleoyl-glycero-3-phosphocholine (POPC) lipids, and the system was solvated in a periodic 120 Å by 120 Å by 140 Å box of explicit water and neutralized with 0.15 M Na^+^ and Cl^−^ ions. We used the Amber ff14SB-ildn (*81*) and lipid 14 (*82*) force fields, as well as the Amber force field parameters, for phosphorylated amino acids (*83*). The TIP3P (*84*) and the Joung-Cheatham (*85*) parameters were used for the water and the ions, respectively. Effective point charges of the phosphoinositide were obtained by RESP fitting (*86*) of the electrostatic potentials calculated with the HF/6-31G* basis set.

After energy minimization, all-atom MD simulations were carried out using Gromacs 5.1 (*87*) patched with the PLUMED 2.3 plugin (*88*). Each system was gradually heated to 310 K and preequilibrated during 10 ns of brute-force MD in the NPT-ensemble. The replica exchange with solute scaling (REST2) (*89*) technique was used to enhance the sampling of the loop regions at the V2R-arrestin interface. A total of 24 replicas were simulated in the NVT ensemble. REST2 is a type of Hamiltonian replica exchange simulation scheme, which performs many replicas of the same MD simulation system simultaneously. The replicas have modified free energy surfaces, in which the barriers are easier to cross than in the original system. By frequently swapping the replicas during the MD, the simulations “travel” on different free energy surfaces and easily visit different conformational zones. Last, only the samples on the original free energy surface are collected. The replicas are artificial and are only used to overcome the energy barriers. REST2, in particular, modifies the free energy surfaces by scaling (reducing) the force constants of the “solute” molecules in the simulation system. In this case, the loop regions at the V2R-arrestin interface were considered as solute—the force constants of their van der Waals, electrostatic, and dihedral terms were subject to scaling—to facilitate their conformational changes. The effective temperatures used here for generating the REST2 scaling factors ranged from 310 to 1000 K, following a distribution calculated with the Patriksson–van der Spoel approach (*90*). Exchange between replicas was attempted every 1000 simulation steps. This setup resulted in an average exchange probability of ~40%. We performed 120 ns × 24 replicas of MD in the NVT ensemble for each system. The first 40 ns were discarded for equilibration. The original unscaled replica (at 310 K effective temperature) was collected and analyzed.
