# Supplementary material for: Structure of the vasopressin hormone–V2 receptor–β-arrestin1 ternary complex
Source: Sci Adv. 2022 Sep 2;8(35):eabo7761. doi: 10.1126/sciadv.abo7761 (PMC10866553; doi:10.1126/sciadv.abo7761)
Supplement: Supplementary file 1 — Figs. S1 to S15 Tables S1 and S2 [file sciadv.abo7761_sm.pdf]

Science Advances
AAAS

Supplementary Materials for

Structure of the vasopressin hormone–V2 receptor–β-arrestin1 ternary complex

Julien Bous *et al.*

Corresponding author: Sébastien Granier, sebastien.granier@igf.cnrs.fr; Bernard Mouillac, bernard.mouillac@igf.cnrs.fr; Patrick Bron, patrick.bron@cbs.cnrs.fr

Sci. Adv. **8**, eabo7761 (2022)
DOI: 10.1126/sciadv.abo7761

The PDF file includes:

Figs. S1 to S15
Tables S1 and S2
Legend for movie S1

Other Supplementary Material for this manuscript includes the following:

Movie S1

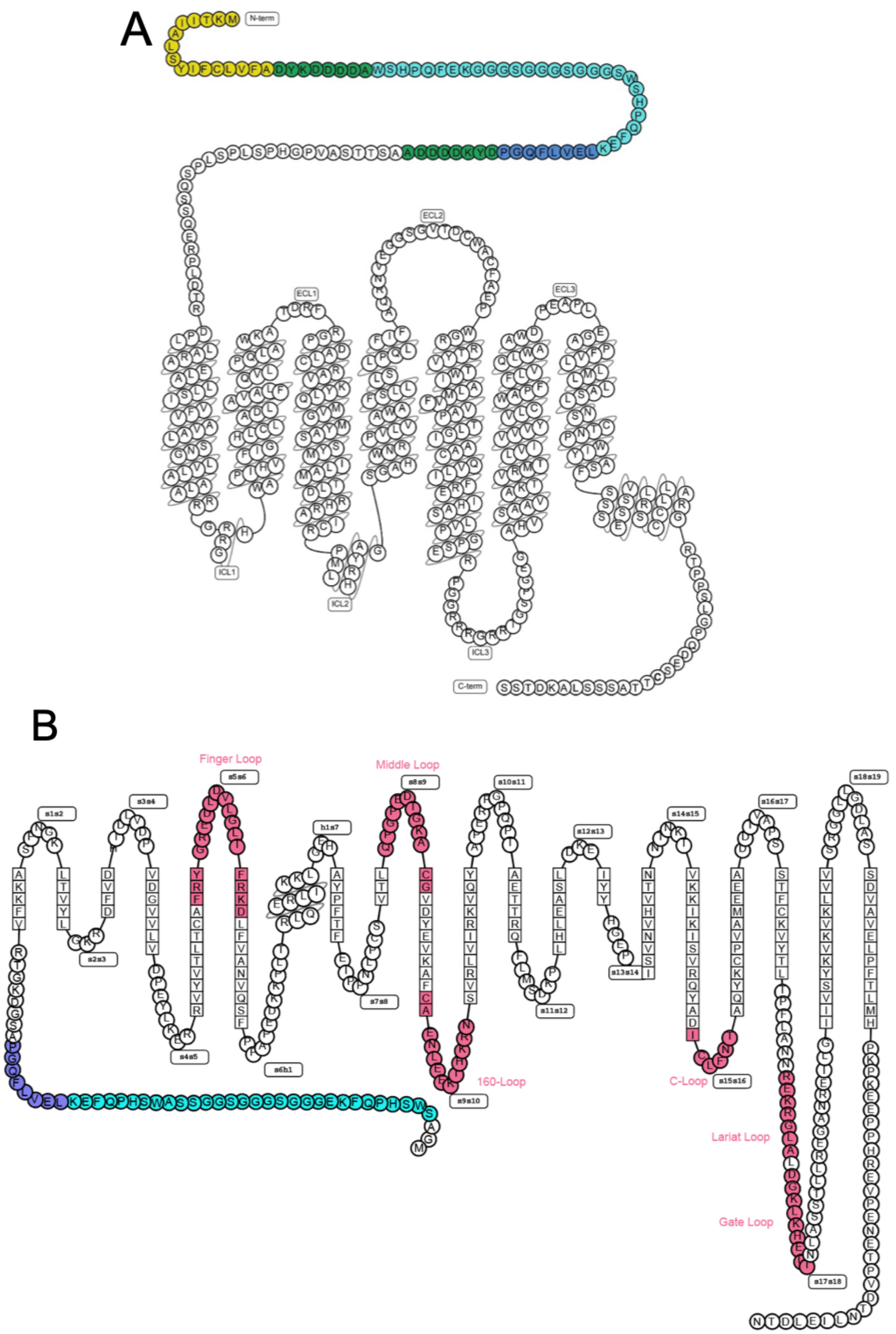
A
N-term
ECL1
ECL2
ECL3
ICL1
ICL2
ICL3
C-term
B
Finger Loop
Middle Loop
160-Loop
C-Loop
Lariat Loop
Gate Loop
s1s2
s2s3
s3s4
s4s5
s5s6
s6h1
h1s7
s7s8
s8s9
s9s10
s10s11
s11s12
s12s13
s13s14
s14s15
s15s16
s16s17
s17s18
s18s19

Fig. S1. Cartoons of the V2R and βarr1ΔCT constructs.
(**A**) and (**B**) Modified snake plots of the engineered V2R and βarr1 versions used for Cryo-EM structure determination, respectively (https://gpcrdb.org). Purification tags were inserted in the N-terminal part of both constructs for different reasons: i) the C-terminus of V2R has to be maintained in a native form for an efficient βarr1 interaction, ii) the C-terminus of βarr1 was truncated at residue 382 (βarr1ΔCT), leading to a constitutively active form enhancing V2R coupling. The hemagglutinin signal peptide is shown in yellow, the Flag-tags are in green, the Twin-Strep-tags are in cyan, the human rhinovirus 3C protease cleavage sites are in purple, and all βarr1ΔCT loops discussed in the manuscript are depicted in pink.

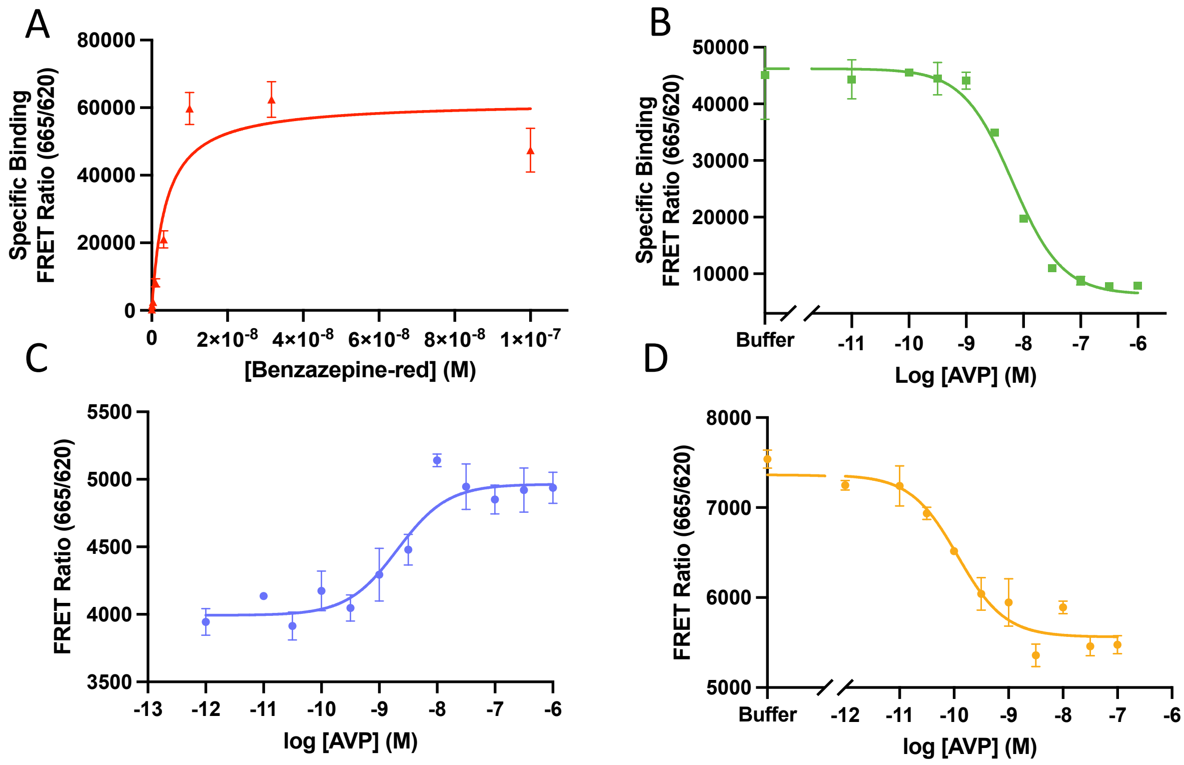

Fig. S2. Pharmacological and functional properties of the V2R construct.
(**A**) Binding of the benzazepine-red fluorescent antagonist to the V2R measured by FRET. Specific binding from a typical saturation assay is shown as FRET ratio (665nm/620nm x 10,000). (**B**) Binding of AVP to the V2R is illustrated as FRET ratio (665nm/620nm x 10,000). Specific binding of the fluorescent antagonist is shown. For each competition curve, it was used at 5 nM with or without increasing concentrations of AVP. (**C**) Dose-response of V2R-dependent recruitment of the βarr2 to AP2 measured by FRET ratio (665nm/620nm x 10,000) in the presence of increasing concentrations of AVP. (**D**) Dose-response of V2R-dependent Gs protein/adenylyl cyclase activation measured by FRET ratio (665nm/620nm x 10,000). The cAMP accumulation which displaces the fluorescently-labeled cAMP binding to its specific antibody is shown in the presence of increasing concentrations of AVP. For each assay, a typical experiment is represented from at least 3 independent experiments, each point performed in triplicate. Each value is presented as mean ± SEM.

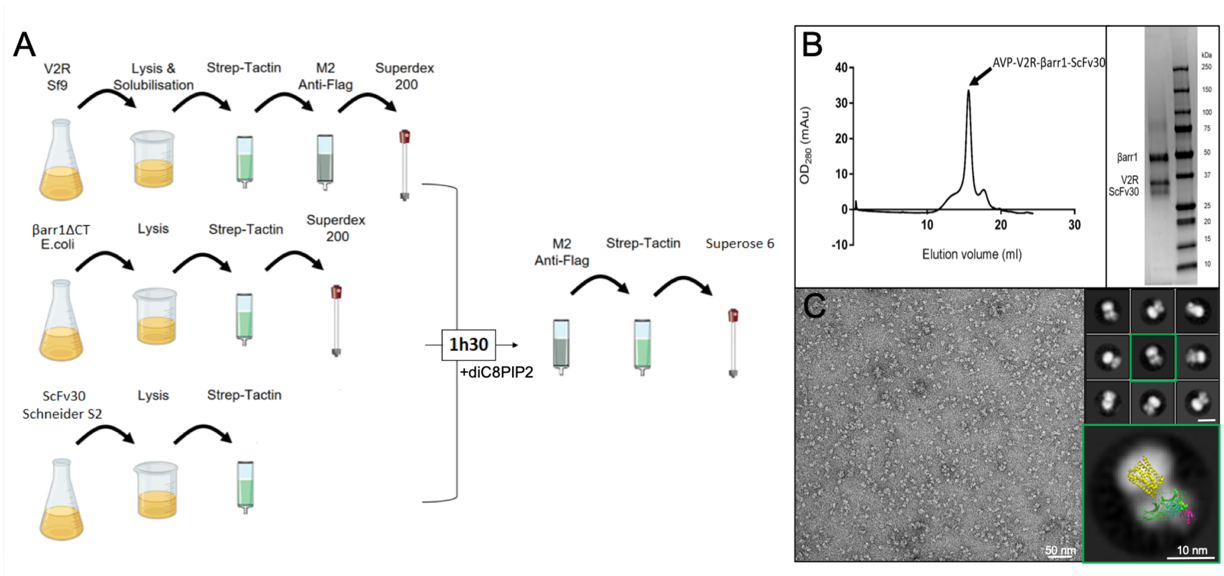

Fig. S3. Overview of the AVP-V2R-βarr1ΔCT-ScFv30 complex preparation, purification and NS-EM analysis.

(**A**) Workflow for AVP-V2R-βarr1ΔCT-ScFv30 assembly. The V2R, βarr1ΔCT and ScFv30 were expressed and purified separately, the complex being incubated for 1h30 in the presence of diC8PIP2 and then isolated by two successive affinity chromatography steps and a final SEC. (**B**) A representative chromatogram of the AVP-V2R-βarr1ΔCT-ScFv30 complex using a Superose 6 column shows a monodisperse peak. Fractions containing the sample were combined, used directly for NS-EM and concentrated for Cryo-EM grid preparation. SDS-PAGE of peak fraction from the Superose 6 step is shown on the right. Coomassie blue staining of the proteins confirmed the presence of βarr1ΔCT, V2R and ScFv30 in the complex (AVP is not visible). (**C**) NS-EM analysis of the sample. A representative micrograph of the purified AVP-V2R-βarr1ΔCT-ScFv30 complex isolated from the SEC peak (scale bar, 50 nm) is shown on the left. 2D most representative class averages showing different orientations (scale bar, 10 nm) are illustrated (top right). A close-up view of a typical 2D class average is presented (bottom right). The 3D model of the M2R-βarr1-Nb24-ScFv30 complex (PDB 6u1n) is fitted onto the particle to show that the AVP-V2R-βarr1ΔCT-ScFv30 particle displayed typical size and characteristics of a GPCR-βarr1-ScFv30 assembly (M2R, yellow; βarr1, green; Nb24, blue; ScFv30, pink).

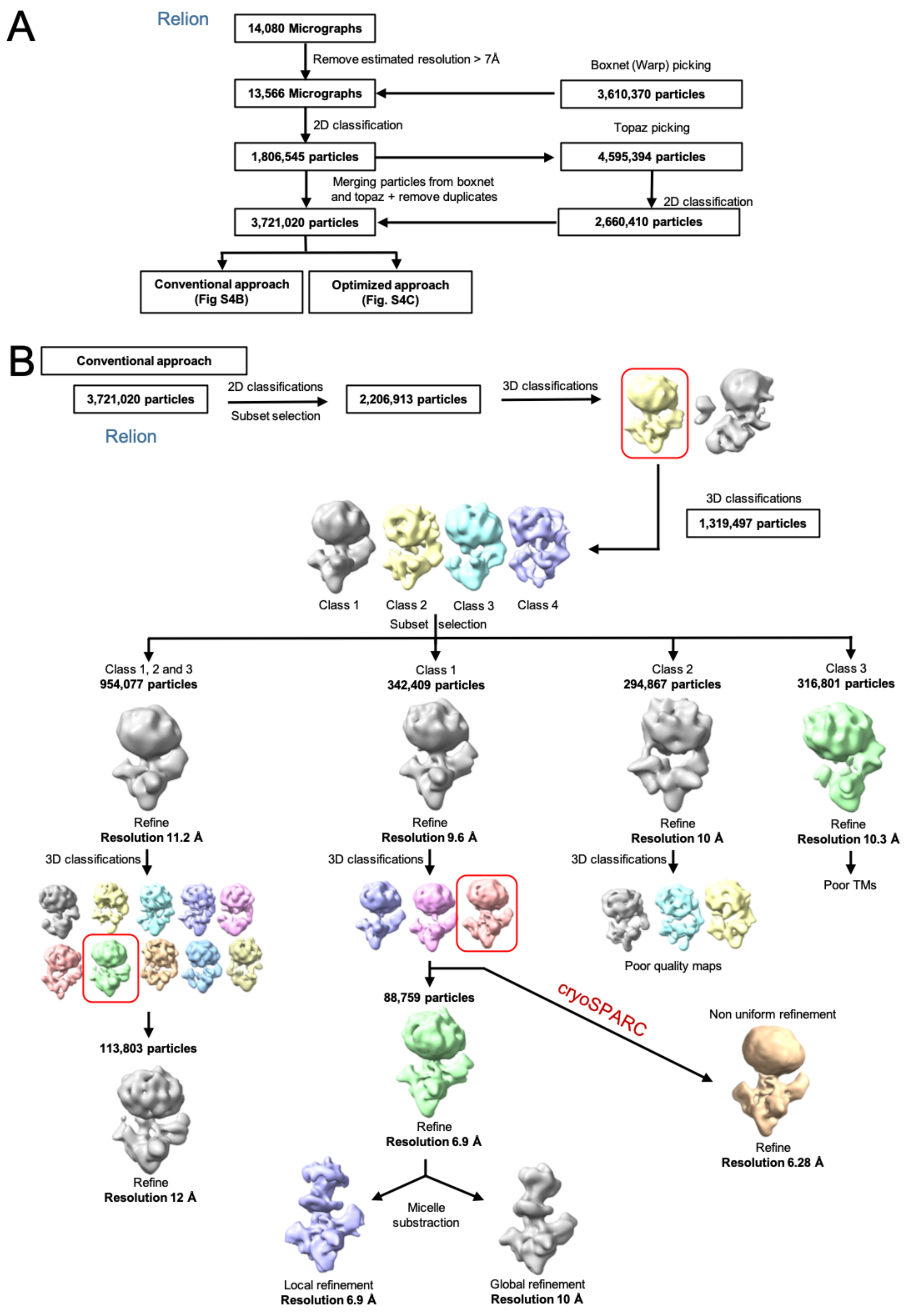
A
Relion
14,080 Micrographs
Remove estimated resolution > 7Å
13,566 Micrographs
Boxnet (Warp) picking
3,610,370 particles
2D classification
Topaz picking
1,806,545 particles
4,595,394 particles
Merging particles from boxnet and topaz + remove duplicates
2D classification
3,721,020 particles
2,660,410 particles
Conventional approach (Fig S4B)
Optimized approach (Fig. S4C)
B
Conventional approach
3,721,020 particles
Relion
2D classifications
Subset selection
2,206,913 particles
3D classifications
3D classifications
1,319,497 particles
Class 1
Class 2
Class 3
Class 4
Subset selection
Class 1, 2 and 3
954,077 particles
Class 1
342,409 particles
Class 2
294,867 particles
Class 3
316,801 particles
Refine
Resolution 11.2 Å
Refine
Resolution 9.6 Å
Refine
Resolution 10 Å
Refine
Resolution 10.3 Å
3D classifications
3D classifications
3D classifications
Poor TMs
Poor quality maps
88,759 particles
cryoSPARC
Non uniform refinement
113,803 particles
Refine
Resolution 6.9 Å
Refine
Resolution 6.28 Å
Refine
Resolution 12 Å
Micelle substraction
Local refinement
Resolution 6.9 Å
Global refinement
Resolution 10 Å

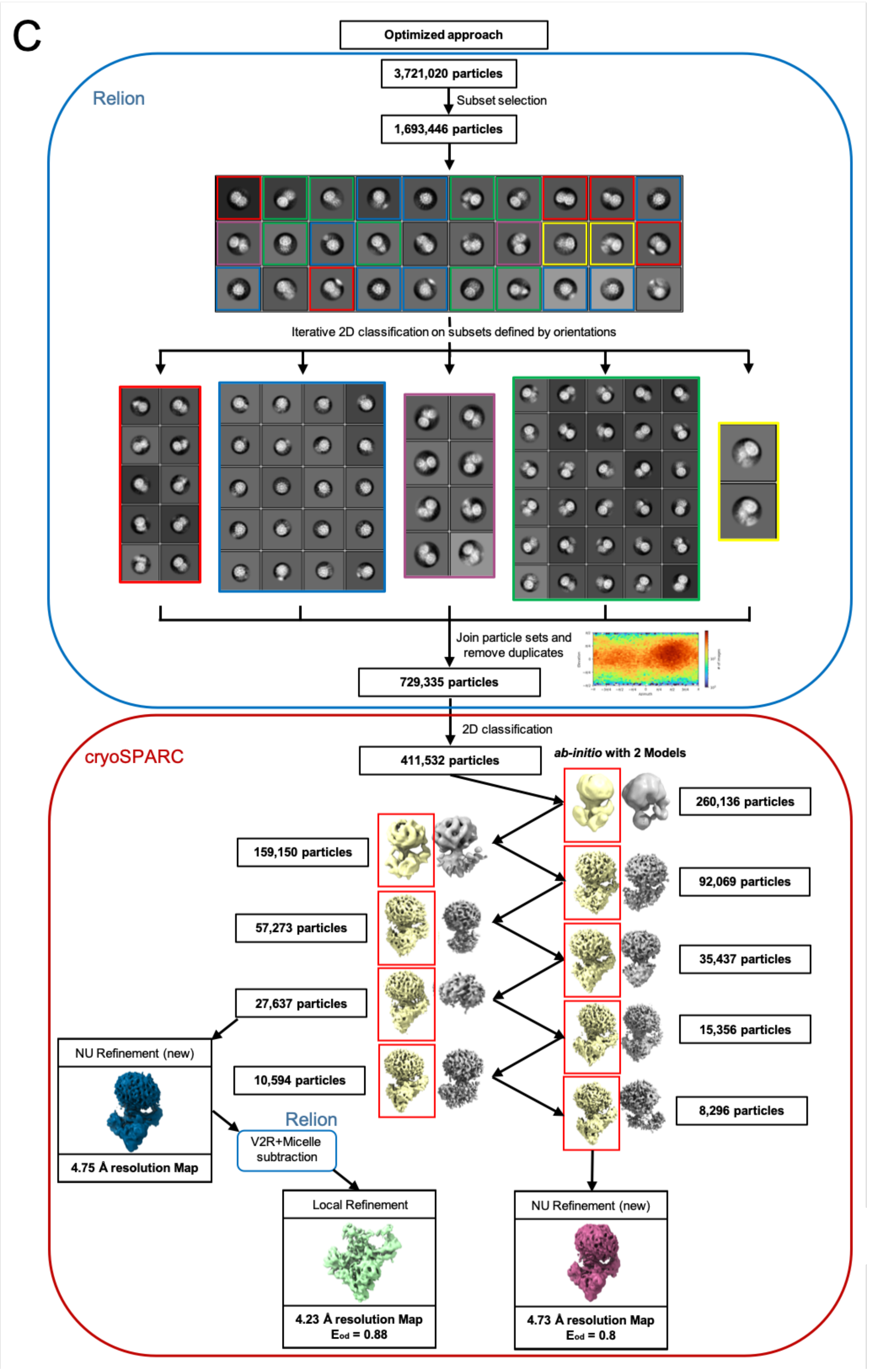

C
Optimized approach
Relion
3,721,020 particles
Subset selection
1,693,446 particles
Iterative 2D classification on subsets defined by orientations
Join particle sets and remove duplicates
729,335 particles
2D classification
cryoSPARC
411,532 particles
ab-initio with 2 Models
260,136 particles
159,150 particles
92,069 particles
57,273 particles
35,437 particles
27,637 particles
15,356 particles
10,594 particles
8,296 particles
NU Refinement (new)
4.75 Å resolution Map
Relion
V2R+Micelle subtraction
Local Refinement
4.23 Å resolution Map
Eod = 0.88
NU Refinement (new)
4.73 Å resolution Map
Eod = 0.8

Fig. S4. Cryo-EM workflow.

The different steps of the single particle analysis from a unique movie dataset collected with a Titan Krios are detailed (see also Materials and Methods). Micrographs were treated (movie selection and particle picking) to screen particles which were first subjected to iterative 2D classification in Relion (**A**). From a set of 3,721,020 particles, two different approaches were performed, a conventional one (**B**) and an optimized one (**C**). The conventional workflow, using a 2D classification (only for selected correctly picked particles) and diverse 3D classification approaches in Relion and cryoSPARC provided 3D reconstructions with no better density than 6.28 Å. The optimized approach, combining multiple 2D classifications (based on the orientation of the particles and using different mask size) in Relion followed by multiple rounds of *ab initio* reconstructing processing steps in cryoSPARC, led to a stack of 27,637 particles which was selected to generate a density map with an overall resolution of 4.75 Å after non-uniform refinement. Subtraction of micelles and V2R and local refinement yielded another map with an overall resolution of 4.23 Å. In addition, the subset of 27,637 particles was also refined using 3 additional *ab initio* steps to obtain another stack of 8,296 particles yielding a density map with an overall resolution of 4.73 Å after NU refinement. The Eod, a coefficient related to the orientation distribution via its corresponding point spread function, calculated from the 4.23 Å and 4.73 Å resolution maps, was significantly above the 0.6 threshold (0.88 and 0.8, respectively).

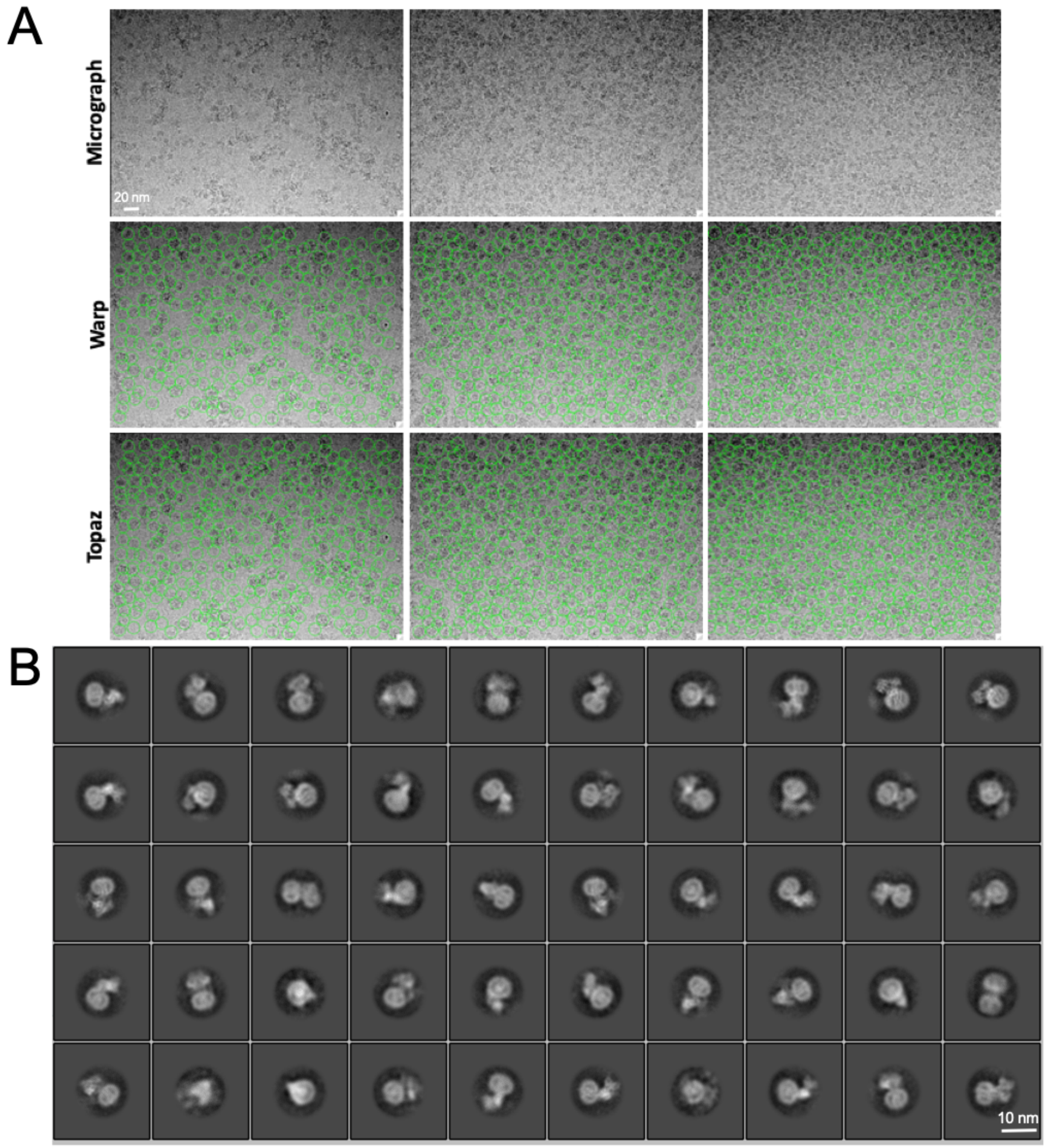

Fig. S5. Cryo-EM images and 2D class averages of the AVP-V2R-βarr1ΔCT-ScFv30 complex.
(**A**) Three representative micrographs of the complex, showing a different distribution of particles in ice (first row). The picking of the particles was done with Warp or Topaz (second and third row, respectively). Each single particle is circled in green. The combination of Warp and Topaz allowed for an optimal recovery of the data. (**B**) Most representative 2D class averages showing distinct secondary structure features like the V2R TM regions embedded in the detergent micelle, and different orientations of the AVP-V2R- βarr1ΔCT-ScFv30 complex.

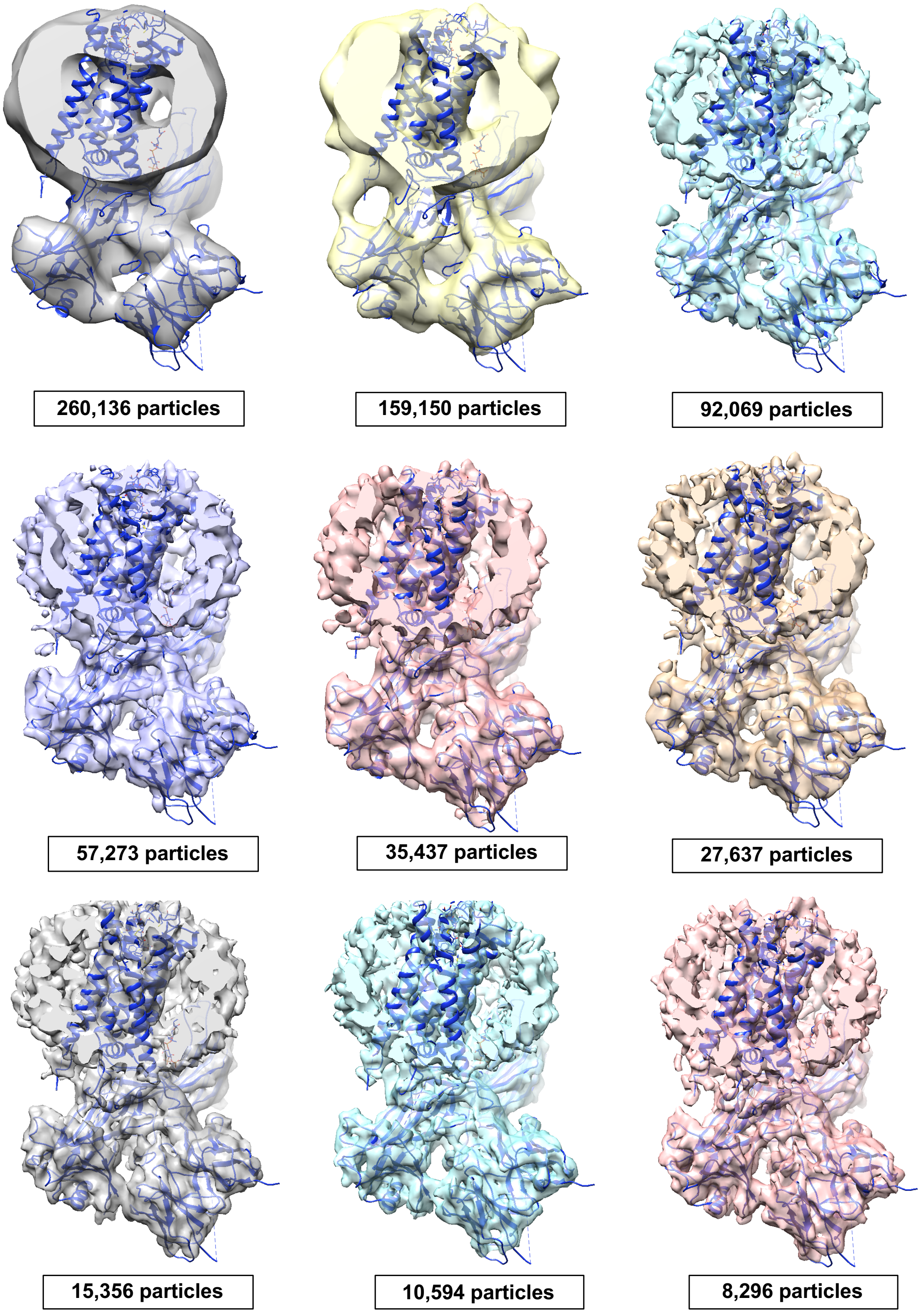

Fig. S6. Screening of the particles with a two-models *ab initio* reconstruction procedure. The refined 3D model fitted in the successive density maps shows that orientation of βarr1ΔCT-ScFv30 relative to V2R is maintained through selected models from the iterative rounds of CryoSPARC *ab initio* analysis.

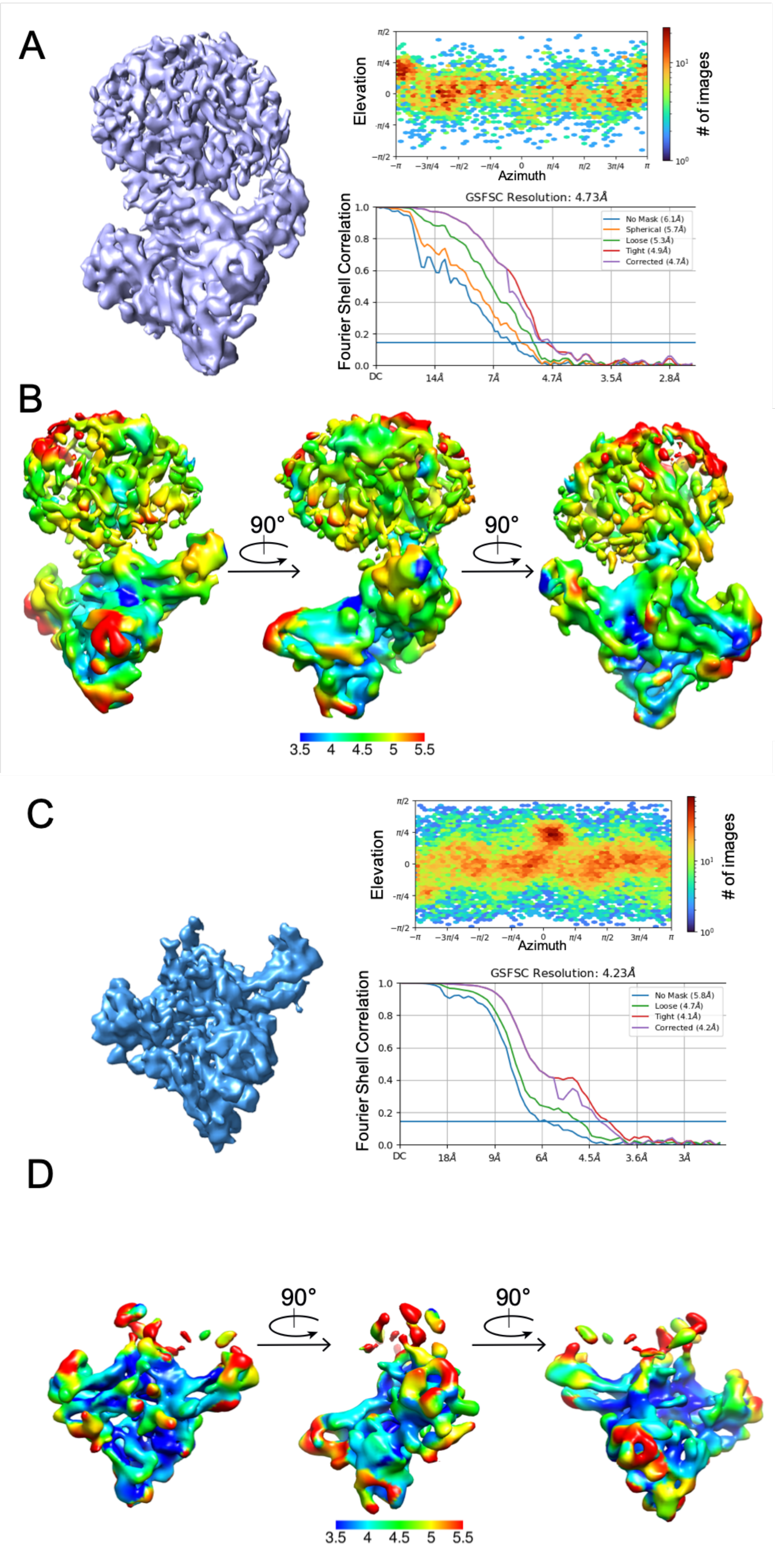

A
Elevation
Azimuth
# of images
GSFSC Resolution: 4.73Å
Fourier Shell Correlation
No Mask (6.1Å)
Spherical (5.7Å)
Loose (5.3Å)
Tight (4.9Å)
Corrected (4.7Å)
DC
14Å
7Å
4.7Å
3.5Å
2.8Å
B
90°
90°
3.5 4 4.5 5 5.5
C
Elevation
Azimuth
# of images
GSFSC Resolution: 4.23Å
Fourier Shell Correlation
No Mask (5.8Å)
Loose (4.7Å)
Tight (4.1Å)
Corrected (4.2Å)
DC
18Å
9Å
6Å
4.5Å
3.6Å
3Å
D
90°
90°
3.5 4 4.5 5 5.5

Fig. S7. Cryo-EM density maps of the AVP-V2R-βarr1ΔCT-ScFv30 complex and local resolution estimation.

(**A**) Final density map of the whole AVP-V2R-βarr1ΔCT-ScFv30 complex and (**B**) its local resolution estimation. (**C**) Final density map of the V2RCter-βarr1ΔCT-ScFv30 subcomplex and (**D**) its local resolution estimation. For both complexes, viewing direction distribution (top panels) indicates that no preferential orientation of the particles was observed. The density map resolution was determined from Fourier Shell Correlation (FSC) with a cut-off of 0.143 (bottom panel).

A

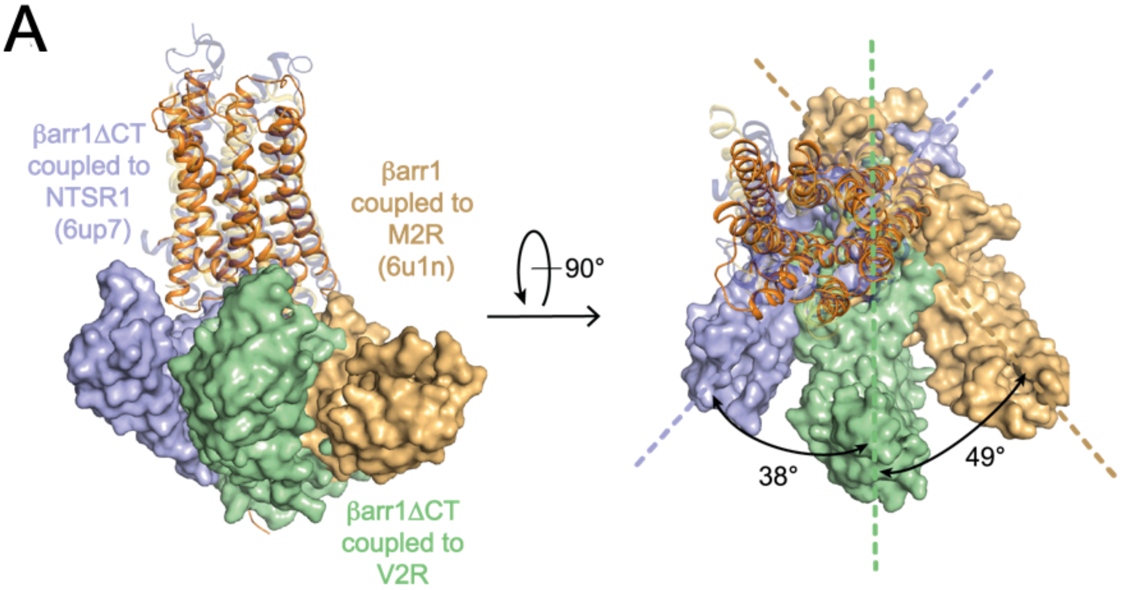

B

	V2R	
	Angle (°)	translation (Å)
**M2R - 6u1n**	49	15
**β1AR - 6tko**	38	8
**NTSR1 - 6up7**	38	14
**NTSR1 - 6pwc**	54	15
**Rhodopsin - 5w0p**	49	11

Fig. S8. Comparison of the βarr1ΔCT orientation in different GPCR complexes.
(**A**) Overlay of the V2R-βarr1ΔCT structure with NTSR1-βarr1ΔCT (PDB 6up7) and M2R-βarr1 (6u1n) structures, on the basis of alignment of the receptor chains, viewed from the membrane (left) and from the extracellular space (right). The orientation of βarr1ΔCT in the V2R complex is compared with that in the NTSR1 and in the M2R complexes respectively, and the angles between βarr1s are indicated. V2R and βarr1ΔCT are colored in orange and green, M2R and βarr1 (PDB 6u1n) are in yellow and gold, βarr1ΔCT and NTSR1 (PDB 6up7) are in purple. (**B**) Geometrical parameters of the different GPCR-arrestin complexes compared to the V2R-βarr1ΔCT complex. The rhodopsin-arrestin1 (PDB 5w0p) is also indicated for information (91). Angles and distances were extracted from Pymol 2.3.5 macro's "angle_between_domains". They represent rotation and displacement that would happen to align βarr1s after each couple of GPCRs is aligned.

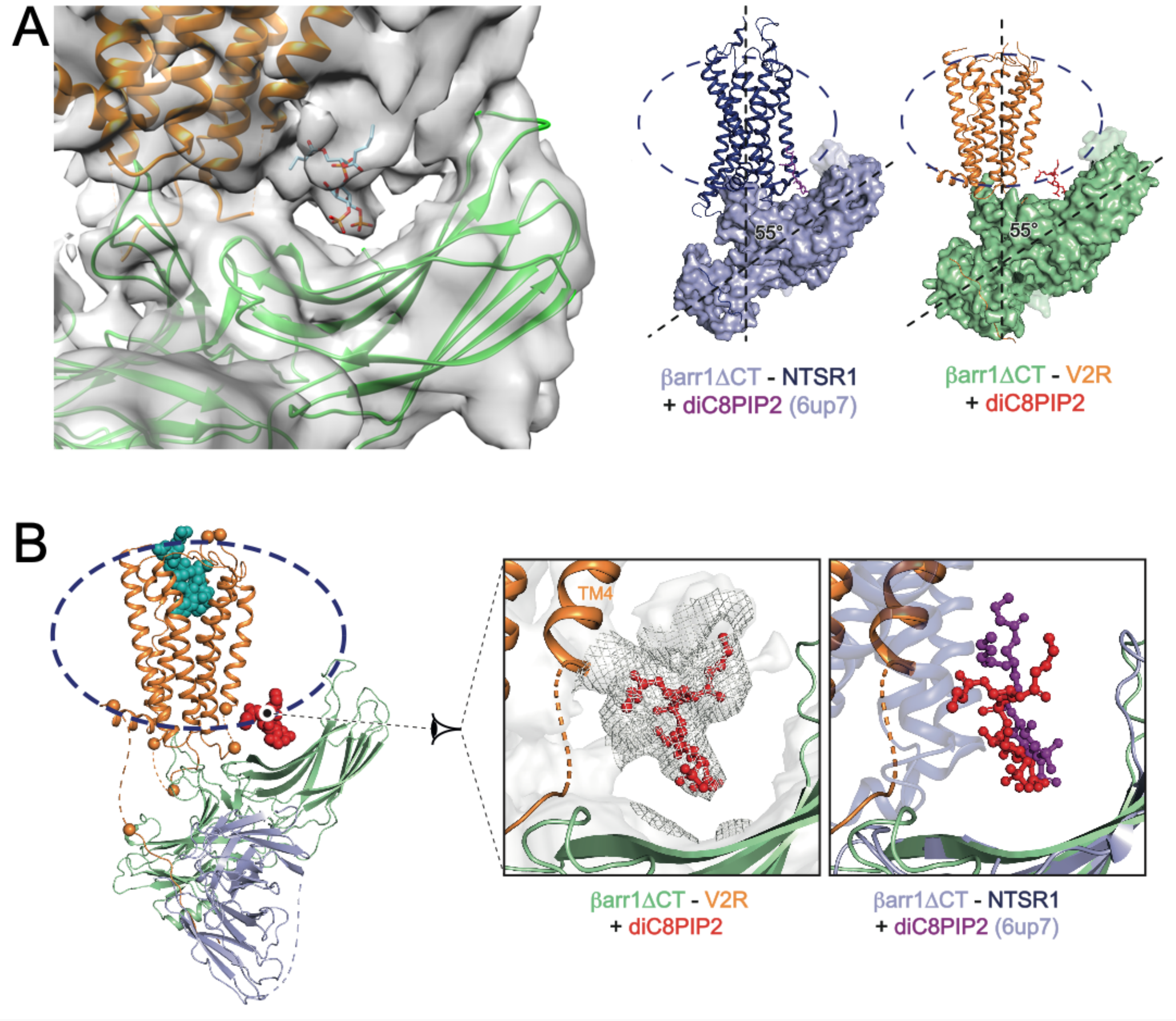

Fig. S9. Putative localization of diC8PIP2 in the AVP-V2R-βarr1ΔCT-ScFv30 complex.

(**A**) Based on the density map of the AVP-V2R-βarr1ΔCT-ScFv30 complex, and on several evidences (see main text), the diC8PIP2 molecule was tentatively modeled at the interface between V2R and the phosphoinositide binding site of βarr1 (left panel). The tilted conformation of βarr1ΔCT in the AVP-V2R-βarr1ΔCT-ScFv30 complex and the $NTS_{8\text{-}13}$-NTSR1-βarr1ΔCT-Fab30 complex (PDB 6up7) were compared (right panel). The detergent micelles are shown as dashed lines. The angle between the longitudinal axis of βarr1 and each GPCR, respectively, is indicated. The diC8PIP2 in V2R and NTSR1 (PDB 6up7) complexes is shown in red and mauve, respectively. NTSR1 is in dark purple whereas βarr1ΔCT is blue grey. (**B**) Putative 3D model of diC8PIP2 in the AVP-V2R-βarr1ΔCT-ScFv30 complex (left panel). A close-up view of the diC8PIP2 position is shown in the central panel. Its putative density is displayed as a mesh. An overlay of V2R-βarr1ΔCT with NTSR1-βarr1ΔCT (right panel) shows that diC8PIP2

superimposes in the two complexes (alignment is done onto βarr1ΔCT moieties). In A and B, the color scheme for V2R and βarr1ΔCT is as in Fig. 1.

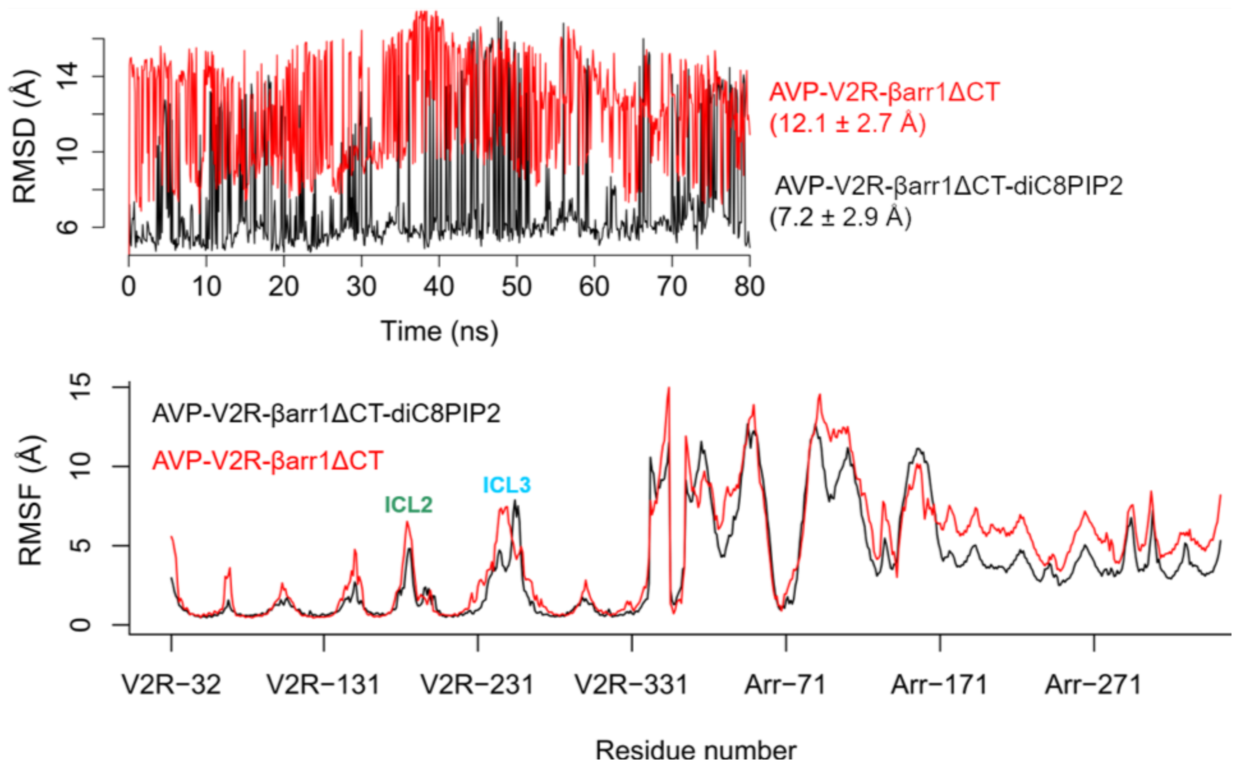

Fig. S10. MD simulations of the AVP-V2R-βarr1ΔCT complex: effect of diC8PIP2.
Root-mean-square deviations (RMSD, top panel) and fluctuations (RMSF, bottom panel) of V2R-βarr1ΔCT Cα atoms, during the MD simulations of the AVP-V2R-βarr1ΔCT complex with or without diC8PIP2. The MD trajectories were aligned to the Cα atoms of V2R TM helices in the cryo-EM structure.

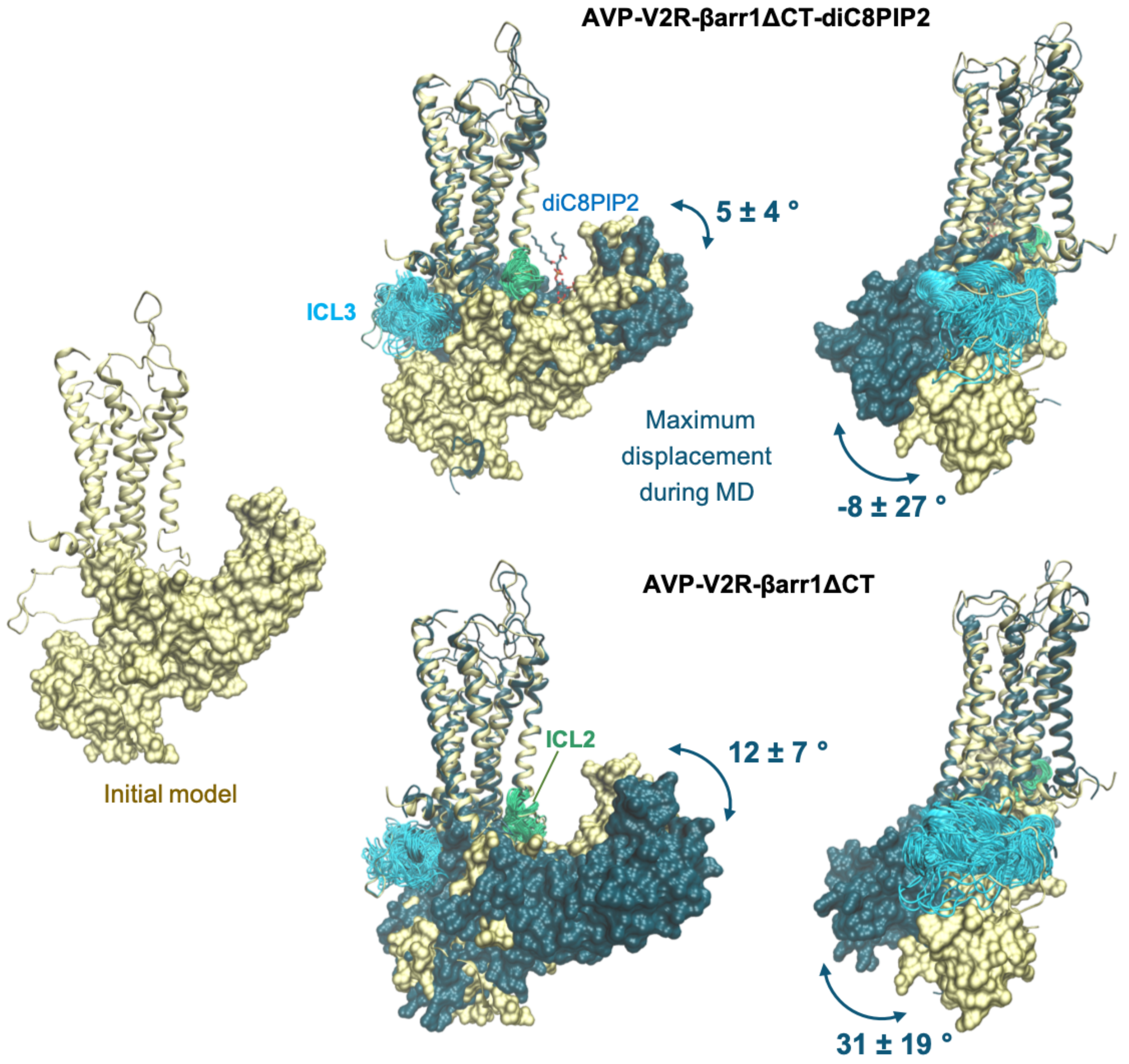

Fig. S11. MD simulations of the AVP-V2R-βarr1ΔCT complex: mobility of V2R ICLs and βarr1ΔCT.

The mobility of V2R ICL2, ICL3 and βArr1ΔCT during the MD simulations of the AVP-V2R-βArr1ΔCT complex with (top) or without (bottom) diC8PIP2 is shown. MD trajectories were aligned to the Cα atoms of V2R TM helices in the initial model built based on the Cryo-EM structure. Rotation of βArr1ΔCT N-lobe is measured as a dihedral angle with respect to the axis of TM2. Tilt of βArr1ΔCT C-lobe is calculated as a dihedral angle with respect to the membrane plane. All angles are calculated relative to the initial model, labeled as mean ± SD during the MD.

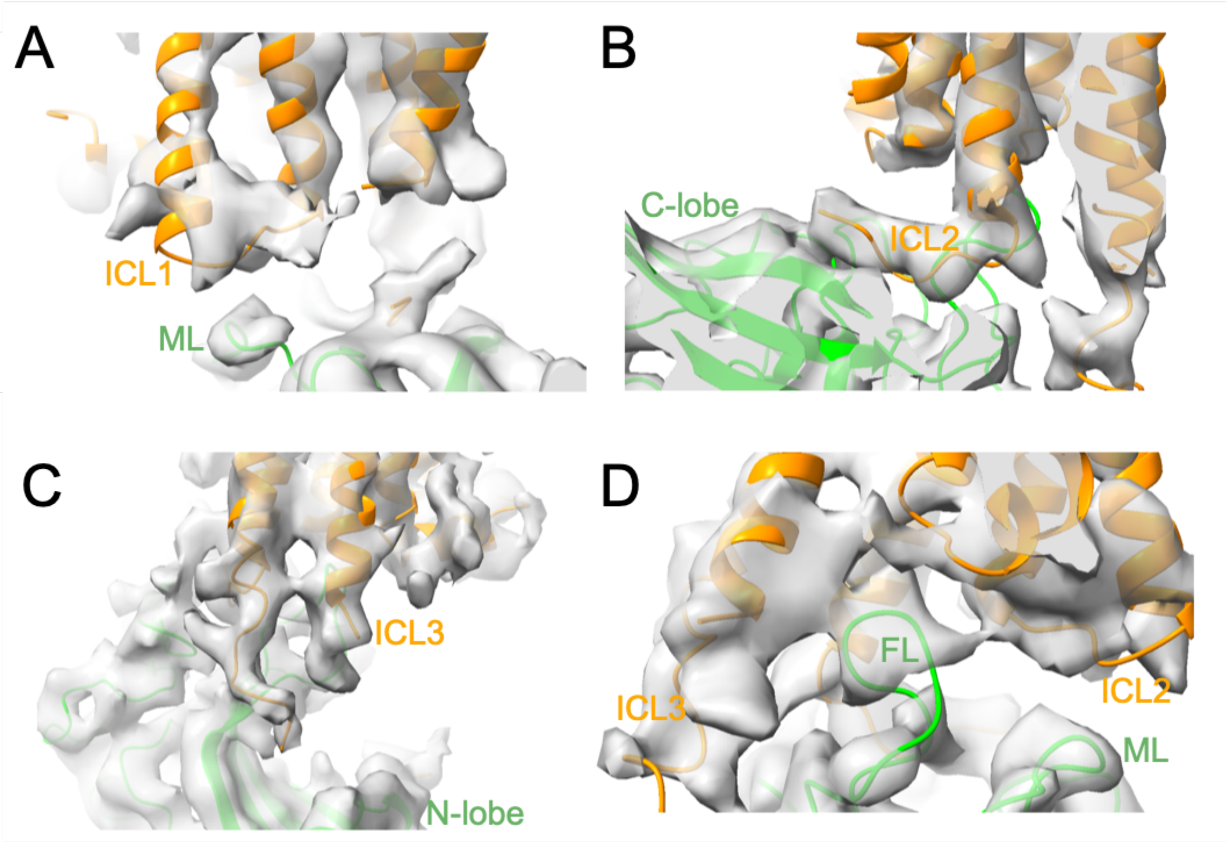

Fig. S12. Interactions at the interface between V2R and βarr1ΔCT.
In all panels, the cryo-EM density map (in grey) and the corresponding 3D model of the V2R-βarr1ΔCT complex are superimposed (V2R in dark orange and βarr1ΔCT in lime green). All intracellular regions of the V2R, ICLs and the cytoplasmic side of the TM core, are in contact with the βarr1ΔCT. Some closed-up views of the interactions at the V2R-βarr1ΔCT interface are shown: ICL1-ML (**A**), ICL2-C-lobe (**B**), ICL3-N-lobe (**C**) and TM core-FL (**D**).

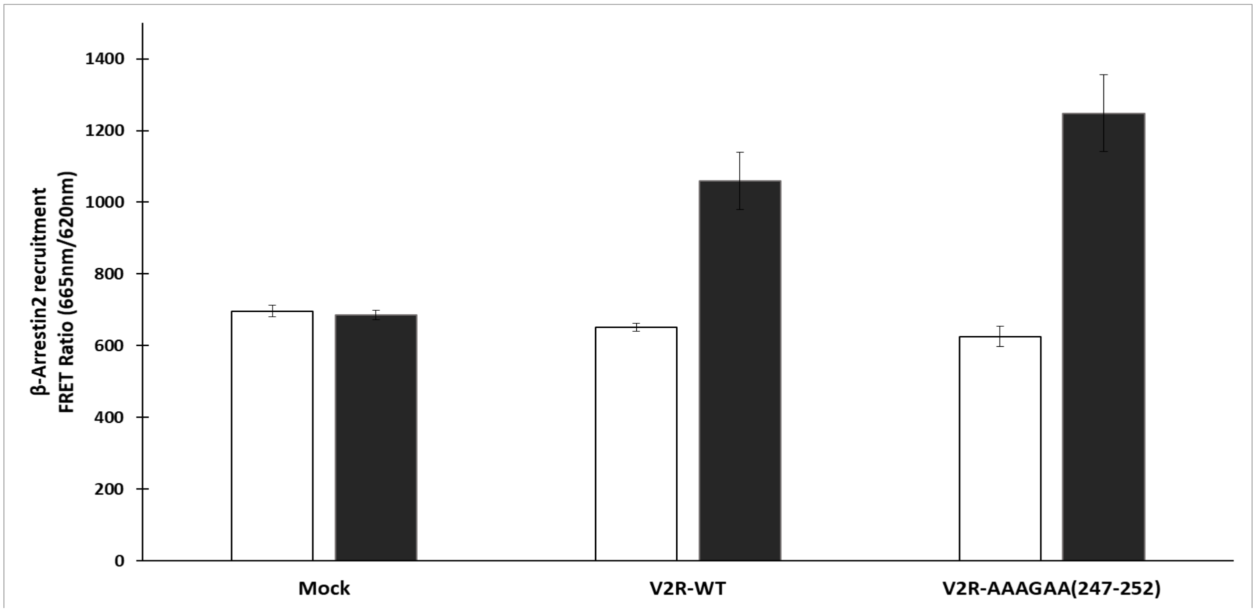

Fig. S13. Mutation of the arginine cluster in V2R ICL3: effect on β-arrestin2 recruitment. HEK293 cells were transfected and agonist-stimulated as described in Materials and Methods. Briefly, 2 ng of V2R coding-plasmid (either the wild-type or the AAAGAA(247-52) mutant) were mixed with 98 ng of non-coding plasmid per each well. Mock cells correspond to cells transfected with the non-coding plasmid only (100 ng). Activation of the cells with AVP (100 nM, black histograms) or buffer (basal, white histograms) was used to measure recruitment of βarr2 and FRET measurements were performed following manufacturer's instructions. A typical experiment is shown, was repeated at least 3 times, each point in triplicate. Each value is expressed as mean ± SEM.

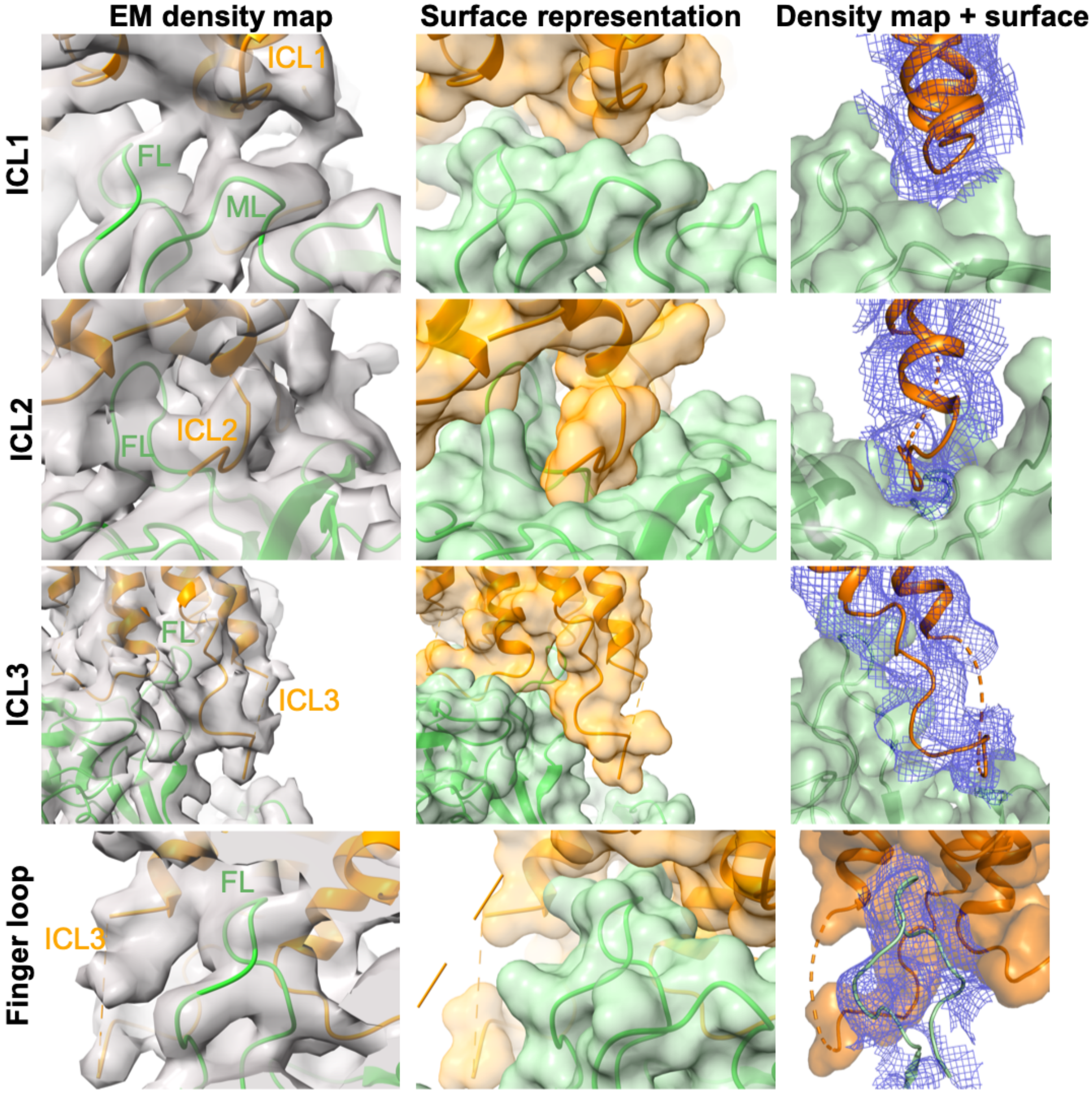

Fig. S14. Close-up views of the interface contacts between V2R and βarr1ΔCT.
The contacts at the V2R-βarr1ΔCT interface are shown in different ways: i) density map (in grey) and corresponding model are superimposed in the left column, ii) surface representation in the middle column, and iii) density map (as a blue mesh) combined with surface representation (two different angles are shown) in the right column. In all panels, V2R is in orange and βarr1ΔCT in green.

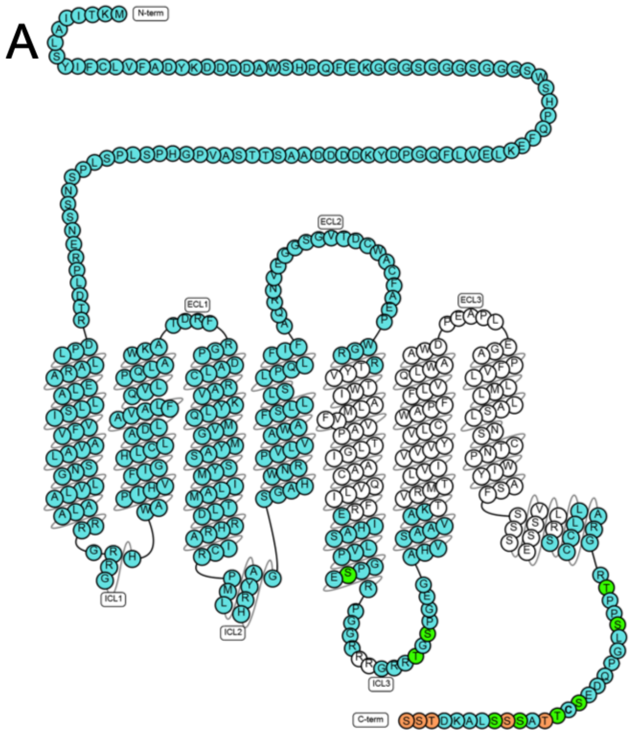

B

		Localization probability	
		**- AVP**	**+ AVP**
**ICL3**	S241	1	0,99
	T253	0,78	0,79
	S255	0,99	0,99
**Cter**	T347	1	0,99
	S350	1	1
	S357	0,99	0,99
	T359	0,83	0,81
	T360	0,67	0,62
	S362	0,82	0,88
	S363	0,45	0,66
	S364	0,94	0,92
	T369	0,27	0,38
	S370	0,73	0,48
	S371	0,55	0,56
		Significant	
		Not significant	

Fig. S15. Phosphoproteomics of the Cryo-EM V2R version.
(**A**) Modified snake plot of the engineered V2R used for cryo-EM structure determination. Suspensions of Sf9 cells expressing the recombinant V2R were treated or not with AVP 1 µM for 30 min at 28°C before harvesting. The V2R was then purified using the procedure described in Materials and Methods, isolated as a monodisperse peak by SEC and subjected to trypsin digestion. Peptides were analyzed using nano-HPLC and tandem mass spectrometry. Identified peptides allowed to recover most (approximately 70%) of the V2R sequence (shown in cyan). Phosphoresidues identified by LC-MS/MS are in green and orange. (**B**) Localization probability of phosphoresidues. Phosphosites were identified in ICL3 and V2RCter regions from cells treated by AVP or not. Significant localization probability (at least 0.75) of phosphates is shown in green, whereas the not significant (< 0.75) is shown in orange.

	AVP-V2R-βArr1ΔCT-ScFv30 (PDB 7R0C; EMD-14221)	V2RCter-βArr1ΔCT-ScFv30 (PDB 7R0J; EMD-14223)
**Data collection and processing**		
Voltage (kV)	300	300
Electron exposure (e-/A2)	52.63	52.63
Defocus range (µm)	-1.0 to -2.0	-1.0 to -2.0
Pixel size (Å)	0.64	0.64
Symmetry imposed	C1	C1
initial particle images (no.)	3,721,020	3,721,020
Final particle images (no.)	8,293	27,637
Map resolution (Å)	4.73	4.23
FSC threshold	0.143	0.143
Map local resolution range (Å)	3.5 to 5.5	3.5 to 5.5
**Refinement**		
Initial models used (PDB codes)	7KH0, 4JQI, 6U1N	4JQI, 6U1N
Model resolution (Å)	4.73	4.23
Map sharpening B factor (Å^2)	-168.2	-188
Model composition		
Non-hydrogen atoms	6,816	4,669
Number of protein residues / atoms	876 / 6,816	596 / 4,669
Number of ligands / ligand atoms	1 / 40	0
average B factor (Å^2)		
Protein	207	164
Ligands	254	–
R.m.s deviations		
Bond lenghts (Å)	0.006	0.008
Bond angles (°)	1.11	1.16
Validation		
MolProbity score	2.32	2.57
Clashscore	26.7	37.3
Poor rotamers (%)	0.14	0.20
Ramachandran plot		
Favored (%)	93.85	91.16
Allowed (%)	6.15	8.84
Disallowed (%)	0	0

Table S1. Cryo-EM data collection, refinement, and validation statistics.
EMD, Electron Microscopy Data; PDB, Protein Data Bank; R.m.s deviations, root mean square deviation.

	V40	V41	L42	E50	R51	R52	V53	Y54	V55	T56	P124	C125	S126	V127	T128	L129	Q130	P131	G132	P133	E134	D135	T136	G137	K138
**AVP-V2R-βarr1ΔCT**																									
G239		5																							
S241		13	1																						
E242																									
R243			7																						
P244		3	9																						
G245		6	1											3											
G246		9	13									3	15	19	8		1		3	1		3	4	1	2
R247	5	18	26					3	7	1	13	1	4	23	9	22	36	9	16	7	3	7	5	3	2
R248			7											1	2	1	1				1	1	1		
R249			3		6	2	4	11	14	3			2	16	3	1	5		4			1	1		
G250				5	8	3	7	17	17	6			1	7	3	2	16	2	16		1	1			
R251					3		1	7	9	1			3	5	6	6									
R252		1	2		1			8	1	1															
T253			1																						
P256			2																						
**AVP-V2R-βarr1ΔCT-diC8PIP2**																									
G246																									
R247		11	2					6	2					2	3	4	19	74	87	33	36				
R248														2		2	6	10	15	4	10				
R249														4	4	4	9	16	50	6	31				
G250								7	11					6	3	3	7	1	4	6	18				
R251								8	7	2											9				
R252														2	1	1			1		9				
T253																					3				

Table S2. Contact map between V2R ICL3 and βArr1ΔCT during MD simulations.
Proposed contacts between residues of V2R ICL3 (row) and βArr1ΔCT (column) by MD simulations of the AVP-V2R-βArr1ΔCT complex in the presence or absence of diC8PIP2. They are colored by the lifetime during the simulations (% of the total simulation frames). A 6 Å minimum distance cutoff between residue pairs was used to calculate the contacts in each MD simulation frame. For instance, in the presence of diC8PIP2, R247 from V2R and G132 from βArr1ΔCT formed the most frequent contact (in 87% of the simulation frames).

Movie S1. Representation of the flexibility of the signaling complex. This animation represents the first three principal components (PC1 to PC3) computed by 3D-variability analysis from cryoSPARC. The strong dynamics of the system was not efficiently managed by the analysis, restricting further interpretation.
